# A Spatiotemporal Characterisation of Redox Molecules in Planarians, with a Focus on the Role of Glutathione during Regeneration

**DOI:** 10.3390/biom11050714

**Published:** 2021-05-11

**Authors:** Karolien Bijnens, Vincent Jaenen, Annelies Wouters, Nathalie Leynen, Nicky Pirotte, Tom Artois, Karen Smeets

**Affiliations:** Centre for Environmental Sciences, Zoology, Biodiversity and Toxicology, Hasselt University, Agoralaan D, 3590 Diepenbeek, Belgium; karolien.bijnens@uhasselt.be (K.B.); vincent.jaenen@uhasselt.be (V.J.); annelies.wouters@uhasselt.be (A.W.); nathalie.leynen@uhasselt.be (N.L.); nickypirotte@hotmail.com (N.P.); tom.artois@uhasselt.be (T.A.)

**Keywords:** planarians, wound healing and tissue regeneration, stem cells, redox molecules, reactive oxygen species, antioxidants

## Abstract

A strict coordination between pro- and antioxidative molecules is needed for normal animal physiology, although their exact function and dynamics during regeneration and development remains largely unknown. Via in vivo imaging, we were able to locate and discriminate between reactive oxygen species (ROS) in real-time during different physiological stages of the highly regenerative planarian *Schmidtea mediterranea*. All ROS signals were strong enough to overcome the detected autofluorescence. Combined with an in situ characterisation and quantification of the transcription of several antioxidant genes, our data showed that the planarian gut and epidermis have a well-equipped redox system. Pharmacological inhibition or RNA interference of either side of the redox balance resulted in alterations in the regeneration process, characterised by decreased blastema sizes and delayed neurodevelopment, thereby affecting tails more than heads. Focusing on glutathione, a central component in the redox balance, we found that it is highly present in planarians and that a significant reduction in glutathione content led to regenerative failure with tissue lesions, characterised by underlying stem cell alterations. This exploratory study indicates that ROS and antioxidants are tightly intertwined and should be studied as a whole to fully comprehend the function of the redox balance in animal physiology.

## 1. Introduction

Reactive oxygen species (ROS) fulfil an important role in animal physiology [1,2]. Historically, they are associated with oxidative stress and cell damage by altering DNA, RNA, proteins and other macromolecules [3]. However, during the last two decades, it became clear that ROS are regulating cell and tissue communication [1,4]. Serving as activators or signal transduction molecules in multiple pathways, they are involved in cell proliferation and survival, differentiation, metabolism and defence strategies [5]. A strong network of antioxidative (AOX) enzymes (e.g., catalase, superoxide dismutase) and metabolites, such as glutathione, keep the redox balance tightly regulated and controlled [6]. The dynamic interplay between AOX and ROS is evolutionary and important [7], but it is often overlooked in scientific research. It is only when redox-related processes are studied as a whole in different conditions and over model systems that new insights in their exact functional role can be obtained.

From this perspective, it was shown that redox molecules play a prominent role in development, wound healing and regeneration [1,2]. The latter is the process in which damaged and lost tissues, organs and even complete body parts are repaired and functionally restored [8]. The degree to which animals are able to regenerate varies across the animal kingdom [9]. It ranges from limited regenerative capacities in adult humans and vertebrates to highly regenerative invertebrates such as *Hydra* and some planarian species, which are able to regenerate almost every part of their body. In between are axolotls, *Xenopus laevis* and *Danio rerio* that can regenerate specific amputated or injured adult tissues and appendages. In several of these organisms [10,11,12], ROS have been described to play a role in rapid wound closure, stem cell activation and blastema formation from which the lost tissue is regrown and functionalised [13,14,15]. Several underlying pathways such as the mitogen-activated protein kinase (MAPK), Notch, Hedgehog and Wnt signalling pathways are activated [16]. As well as ROS, their dynamic interplay with the antioxidative side of the redox balance is also important, illustrated by the observation that vitamin C promotes regeneration in the planarian *Girardia tigrina* [17] and that an extract with antioxidative properties accelerates fin regeneration in zebrafish [18]. In light of evolutionary aspects on how ROS play a role in the development of complex life, or in light of new breakthroughs in regenerative medicine that searches for a way to heal or replace cells and tissues, a proper identification and understanding of the exact regulation and dynamics of the pro-oxidative and antioxidative signals is crucial for future applications in clinical practice.

In this work, we characterised both sides of the redox balance and their role in the regeneration process of the freshwater planarian *Schmidtea mediterranea*. Due to the presence of a large pool of pluripotent stem cells (neoblasts), this flatworm is able to fully restore all its damaged cells and body parts, including the central nervous system, in only seven days, making them ideal organisms to study the role of redox molecules in tissue repair and regeneration [19,20,21]. We addressed several of the above-mentioned knowledge gaps and investigated the temporal and spatial dynamics of pro-oxidative and antioxidative molecules as part of the redox balance, in different physiological states. In addition, we further elucidated their functional roles in the regeneration process, with a specific focus on the antioxidant glutathione.

## 2. Materials and Methods

### 2.1. Planarian Cultivation and Amputation

An asexual strain of the planarian *S. mediterranea* was cultivated in freshwater medium consisting of 1.6 mM NaCl, 1 mM CaCl_2_, 1 mM MgSO_4_, 0.1 mM MgCl_2_, 0.1 mM KCl and 1.2 mM NaHCO_3_ in milliQ water [22]. The worms were fed once a week with veal liver and kept in the dark at a constant temperature of 20° C. Worms of similar sizes (approximately 3–5 mm) were selected for experiments and starved at least seven days before experimental procedures. To study the regeneration process, worms were cut transversely and anterior to the pharynx to obtain a regenerating head and tail part. Regenerative stadia are indicated by dpa (days post amputation).

### 2.2. Exposures to Redox-Influencing Compounds

In total, five different compounds were used that are thought to influence the redox balance. Diphenyleneiodonium chloride (DPI, Sigma-Aldrich, Cat. No. D2926, St. Louis, MO, USA) is a nonspecific flavoprotein inhibitor that interferes with many different electron transporters [23,24], while apocynin (APO, 4′-hydroxy-3′-methoxy-acetophenone, Sigma-Aldrich, Cat. No. A10809) inhibits NOX (NADPH oxidase) enzymes, acting on the translocation of the cytoplasmic subunits of the enzymes [24,25]. Di-ethyl dithio-carbamate (DETC, Sigma-Aldrich, Cat. No. 228680) is commonly used as superoxide dismutase (SOD) inhibitor [26,27]. Buthionine sulphoximine (BSO, Sigma-Aldrich, Cat. No. B2640) blocks γ-glutamylcysteine synthetase (γ-GS), the rate-limiting step in the production of glutathione [28], while dimethyl fumarate (DMF, Acros-Organics, Cat. No. 222180250, Geel, Belgium) additionally depletes the glutathione pool [29]. In Table 1, the compounds are listed together with their intended targets, as well as their known off-target effects. Stock solutions of 1 mM DETC and 500 mM BSO were prepared in milliQ water, while 3 mM DPI, 4 M APO and 250 mM DMF stock solutions were prepared in 100% dimethyl sulfoxide (DMSO). Final exposure solutions were prepared by diluting the stock solutions in planarian medium. For DPI, APO and DMF, the final exposure concentration of DMSO in the exposure solutions was below the reported threshold of toxicity [30]. Per worm, 1 mL exposure solution was added, and in each experiment, a corresponding condition with medium only was included as a non-exposed control. Every 2–3 days, the exposure solutions were refreshed.

In the first set of exposure experiments, the intact worms were amputated in head and tail fragments that were directly exposed for seven days to planarian medium containing 0.35 µM DETC, 5 mM BSO, 3 µM DPI or 400 µM APO. The used concentrations of DETC and BSO were based on initial screening effects that induce a phenotype, without inducing direct lethality, while the concentrations of DPI and APO were based on a screening by Pirotte et al. 2015 [22]. In a second set of experiments, the antioxidant glutathione was studied in more depth. Therefore, the glutathione content was further depleted by using DMF. Intact worms were pre-exposed for three days to a planarian medium containing 5 mM BSO together with 2.5 µM DMF. Following amputation in head and tail fragments, the worms were exposed again in the glutathione-interfering solution. The timing of the pre-exposure and used concentration of DMF were determined based on an initial screening that showed a significant depletion of the GSH pool without inducing direct lethal effects.

### 2.3. Knockdown of Antioxidative Genes via RNA Interference

RNA interference was performed using double stranded RNA (dsRNA) probes, generated by an in vitro transcription system (T7 RibomaxTM Express RNAi System, Promega, Madison, WI, USA) as indicated by the manufacturer’s instructions. The sequences of the primers are summarized in Supplemental Material S2. Intact animals were injected with three 32.2 nL injections of 1000 ng/µL dsRNA for three consecutive days using the Nanoject II (Drummond Scientific, Broomall, PA, USA). A corresponding control group was injected with milliQ water. The following day, animals were transversally cut in head and tail fragments and allowed to regenerate over seven days. The percentage of downregulation of the target genes was determined by qPCR.

### 2.4. Phenotypic Screening and Blastema Size Determination

For both the exposure experiments as well as the RNAi experiments, the amputated worms were allowed to regenerate for seven days and were observed daily for phenotypic abnormalities (behavioural changes, blister formation, tissue lesions), focusing on regenerative success, blastema formation and development of photoreceptors (eyes). The blastema is an unpigmented region that is formed at the wound site, from which new tissue structures differentiate. The development of photoreceptors was scored 6 dpa or 7 dpa as normal, faint or absent. Pictures were taken with a Nikon DS-Ri2 digital camera mounted on a Nikon SMZ800 stereomicroscope. Sizes of the blastemas were determined using Fiji/ImageJ (version 2.0.0-rc-54/1.51 h) [37] and normalised against the total body area of the worm.

### 2.5. In Vivo Detection of General ROS, Superoxide and Hydrogen Peroxide Production

The compound 5-(and-6)-carboxy-2′,7′-dichlorodihydrofluorescein diacetate (carboxy-H_2_DCFDA, Image-iT LIVE Green Reactive Oxygen Species Detection Kit, Molecular Probes; Invitrogen, I36007, Carlsbad, CA, USA) was used to visualise the general in vivo production of ROS. Superoxide Detection Reagent, Orange (ROS ID superoxide detection kit, Enzo Life Sciences, ENZ-51012, Brussels, Belgium), was used in order to visualise in vivo production of superoxide. Finally, the compound 2′,3′,6′,7′-Tetrahydro-12′-(4,4,5,5-tetramethyl-1,3,2-dioxaborolan-2-yl)-spiro[isobenzofuran-1(3H) or Peroxy Orange 1 (PO1, Sigma-Aldrich, SML0688, St. Louis, MO, USA) was used to specifically stain in vivo hydrogen peroxide.

All three ROS visualisation procedures were performed on intact animals as well as animals with a healing (H) or regenerative (R) wound. Treatment with DPI (which leads to ROS depletion) was combined with staining in order to act as an additional (negative) control (Figure S4B). Depending on the radicals that were stained, animals were exposed to carboxy-H_2_DCFDA (25 μM; general ROS), Orange (0.5 μM; superoxide) or Peroxy Orange 1 (20 μM; hydrogen peroxide) for 1 h prior to wounding (or 1 h prior to imaging in case of the intact animals). Wounded animals were again incubated in the detection reagent for 15 min. Next, worms were gently rinsed with ice-cold medium followed by immobilization and imaging using the Nikon Ds-Ri2 camera placed on a Nikon eclipse 80i fluorescence microscope. In order to fully check the animal’s body on ROS, superoxide or hydrogen peroxide producing structures, the worms were extensively examined dorsally and ventrally. Therefore, intact animals were immobilized in both ways.

Additionally, worms were also visualised using the same experimental setup without incubation with the detection reagent in order to detect possible autofluorescence in the worm (Figure S4A).

### 2.6. Whole-Mount (Fluorescent) In Situ Hybridisation

The expression of *Cu-Zn-sod*, *Mn-sod*, catalase (*cat*), glutathione reductase (*gr*), glutathione-S-transferase (*gst*) and thioredoxin (*trx*) was monitored by a colorimetric in situ hybridisation based on NBT/BCIP (Roche, Basel, Switserland) chemistry, as described by Pirotte et al. 2015 [22]. The expression of the general stem cell marker *smedwi-1* [38] and early progeny marker (committed to the epidermal cell lineage) *NB.21.11e* [39] was determined using whole-mount fluorescent in situ hybridisation (FISH), as described by King and Newmark, 2013 [40]. Probes were synthesized using the DIG RNA (SP6/T7) Labelling Kit (Roche) as indicated by the manufacturer, starting from a purified PCR product of the gene of interest. For this, a general PCR was performed with gene-specific primers with the specific sequences that are summarised in Supplemental Material S2. After mounting in ImmuMount (Thermo Fisher Scientific, Waltham, MA, USA), pictures were taken using a Nikon Ds-Ri2 camera placed on a Nikon eclipse i80 fluorescence microscope. Within one experiment, the imaging settings were kept constant. For *smedwi-1* stains, the average fluorescence intensity per fragment was determined using Fiji/ImageJ. The number of cells expressing *NB.21.11e* were determined in head and tail fragments. For heads, the mid-head region was used, while for tails, the post-pharyngeal area was used to calculate an average number of cells per animal. Cells were counted using Nikon imaging software (NIS-Br) and normalised against the surface of the selected surface area.

### 2.7. Whole-Mount Immunohistochemistry

Mitotically active stem cells were stained by whole-mount immunohistochemistry, as described previously [41]. Therefore, the primary antibody against phosphorylated Histone-H3 (Ser10) (D2C8, rabbit mAb, Cell-Signalling, Danvers, MA, USA, Cat. No. 3377S, diluted 1:1000) was used, together with the secondary anti-rabbit-IgG Alexa Fluor 568-conjugated antibody (Thermo Fisher Scientific, Cat. No. A-11036, diluted 1:500, 3 h at room temperature). After mounting in ImmuMount (Thermo Fisher Scientific), pictures of the samples were taken using a Nikon Ds-Ri2 camera placed on a Nikon eclipse i80 fluorescence microscope. Within one experiment, the imaging settings were kept constant. The total number of mitotic stem cells was counted using Fiji/ImageJ and normalised against the total body size of the worm. In addition, the central nervous system was stained by anti-synapsin 3C11 antibody (Developmental Studies Hybridoma Bank, Iowa, IA, USA, developed by Buchner E.) as described previously [22].

### 2.8. Quantification of Glutathione Content

To measure the total glutathione (GSH) content, a spectrophotometric method was used, based on the recycling assay originally described by Tietze 1969 [42]. Each worm was photographed to determine the mean total body size (mm^2^) per sample for normalisation. Then, 3–5 worms per sample were snap-frozen in liquid nitrogen. To each sample, 213 µL of 200 mM HCl was added, as well as 5 glass beads. The samples were then shredded in cooled holders for 2 min at 30 Hz and kept on ice for the whole procedure. Subsequently, 187 µL was transferred to a new tube, and 10 µL NaH_2_PO_4_ (200 mM, pH 5.6) was added. After an initial addition of 153 µL 200 mM NaOH, the sample was quickly spun down and transferred (330 µL) to a new tube to remove mucus and was then brought to a final pH ranging between 5 and 6. A 100 µM GSH (L-GSH, reduced, Sigma-Aldrich, Cat. No. G6529) stock solution was prepared in 200 mM NaH_2_PO_4_ (pH 5.6) and used to make a two-fold dilution series starting from 2000 pmol as standard. Per reaction, 140 µL mastermix, containing 87.5 µL 200 mM Na_2_PO_4_ with 10 mM EDTA (pH 7.5), 35 µL milliQ water, 8.75 µL 10 mM NADPH, 8.75 µL 12 mM DTNB and 8.225 µL glutathione reductase (GR, Sigma-Aldrich, Cat. No. G3664, 20 U/mL in 200 mM NaH_2_PO_4_-EDTA (pH 7.5)) was added to 60 µL of each standard (in duplicate) or sample (in triplicate) into a 96 well-plate. A no GR control was measured (mastermix without GR) to verify that no signal amplification is present in the absence of GR. A negative control with milliQ water was used as a blank measurement. The total GSH content was determined kinetically at 412 nm with a Fluostar Omega multi-mode microplate reader (30 cycles, 15 flashes per well and cycle, path-length correction 200 µL, 5.88 mm) (BMG Labtech, Ortenberg, Germany). Selecting the first 300 s of the measurement, the slope (∆Absorbance/∆Time) was determined for each blank-corrected sample and standard. The slopes of the standards were used to determine a standard curve and an average slope/pmol per concentration, which, in its turn, was used to determine the pmol of total GSH present per well. This value was corrected for the additional dilutions that were done for pH adjustments and then normalised using the total body surface of the worms per sample, resulting in a total GSH content per mm^2^. To localise the GSH content after mucus removal and interference with stem cells, we treated the worms with N-acetyl-L-cysteine or interfered with *H2B* expression via KD or irradiated the worms with 100 Gray.

### 2.9. Quantification of Superoxide Dismutase Activity

To determine the level of superoxide dismutase (SOD) activity after 24 h of DETC exposure, the reduction of nitroblue tetrazolium (NBT) in the presence of superoxide radicals into diformazan was quantified as originally described by Beauchamp and Fridovich 1971 [43]. For this, 5 worms were pooled per sample and snap-frozen in liquid nitrogen. To each sample, 50 µL ice-cold homogenisation buffer (consisting of 0.1 M Tris-HCl (pH 7.8) with 1 mM EDTA and 1 mM DTT) was added, together with 5 glass beads. The samples were then shredded in cooled holders for 2 min at 30 Hz, followed by a centrifugation at maximum speed for 10 min at 4 °C and kept on ice for the whole procedure. Per reaction, 180 µL mastermix, containing 90 µL 100 mM potassium phosphate buffer (pH 7.8), 68.92 µL milliQ water, 18 µL 130 mM methionine, 0.18 µL 2 mM riboflavin, 1.10 µL 12.23 mM NBT and 1.8 µL 10 mM EDTA, was added to 20 µL of supernatant in a 96 well-plate. As a positive control, 200 µL of the mastermix without supernatant was included. As a blank measurement 200 µL homogenisation buffer was used. To initiate the formation of superoxide radicals, the plate was exposed to a cold light source (3000 K) for 20 min. Then, as a negative control, 200 µL mastermix that was kept in the dark was pipetted into the plate, and the absorbance at 560 nm of all samples were measured with a Fluostar Omega multi-mode microplate reader (path–length correction 200 µL, 5.88 mm) (BMG Labtech). After subtraction of the blank, the absorbance levels were converted to SOD activity (units per ml). One unit of SOD was defined as the enzyme causing 50% inhibition of diformazan formation. Therefore, the absorbance of each sample was subtracted from the average absorbance of the positive control and divided by 50% of the average absorbance of the positive controls. To take the size of the worms into account, the resulting SOD activity was corrected by protein levels (determined on the remaining supernatant via a classical Bradford assay). Finally, the levels were expressed relatively compared to the control.

### 2.10. Gene Expression Analyses

To check the knockdown after RNA interference, one animal per sample was snap-frozen in liquid nitrogen. To study the temporal expression of AOXs, per sample, 5 blastemas were isolated 30 min, 1 h, 3 h or 24 h after amputation in head, trunk and tail fragments. RNA extraction and subsequent quantitative real-time PCR (qPCR) were performed as described previously [41]. Briefly, to extract RNA, a standard phenol:chloroform protocol was followed. First, the snap-frozen samples were dissolved in 100 µL of lysis buffer (Qiagen, Cat. No. 79216, Hilden, Germany) containing 1% β-mercapto-ethanol. The extracted RNA was precipitated using sodium acetate and ethanol. Subsequently, the concentration and purity were determined using the Nanodrop ND-1000. Genomic DNA was removed with the Turbo DNA-free kit (Invitrogen, cat-no AM1907) following the manufacturer’s instructions. Subsequently, cDNA was prepared using the Superscript III first strand synthesis supermix for qRT-PCR (Thermo Fisher Scientific, Cat. No. 11752250). The samples were diluted (1/9) and measured under universal cycling conditions with QuantStudio 5 (Thermo Fisher Scientific) (RNAi knockdown quantification) or an ABI PRISM 7500 platform (Thermo Fisher Scientific) (temporal AOX expression). The most stable reference genes were selected using the geNorm algorithm [44] in qbase^+^ (biogazelle, Ghent, Belgium). Primer sequences are listed in Supplemental Material S2, Tables S1–S3.

### 2.11. Expression Analyses from Publicly Available Data

Using the PlanMine (v3.0, MPI-CBG, Dresden, Germany) database [45], publicly available information regarding the spatial, cell-type specific and temporal gene expression patterns of several antioxidants was consulted in September 2020. For each transcript, the search was restricted to the organism *S. mediterranea* and the dataset dd_smed_v6 and summarized in Supplemental Material S1, Excel file S1.

### 2.12. Statistical Analysis

The blastema sizes, stem cell counts, fluorescent intensities, glutathione content and gene expression data were analysed in RStudio 1.2.1335, R version 3.5.3 (R Core Team, 2019, R Foundation for Statistical Computing, Vienna, Austria). Hypothesis testing was done using analysis of variance (ANOVA) and the Tukey Honest Significant Differences method (Tukey HSD). A normal distribution was assessed using the Shapiro-Wilk test, while homoscedasticity was evaluated using the Bartlett test. When assumptions of normality and homoscedasticity were not met, a Kruskal-Wallis Rank Sum test and a Wilcoxon Rank Sum test were performed. A Bonferroni-correction was used to correct for multiple testing. The sample size of each experiment is indicated in the corresponding figures.

### 2.13. Generation of Graphs and Figures

**Figure 1:**
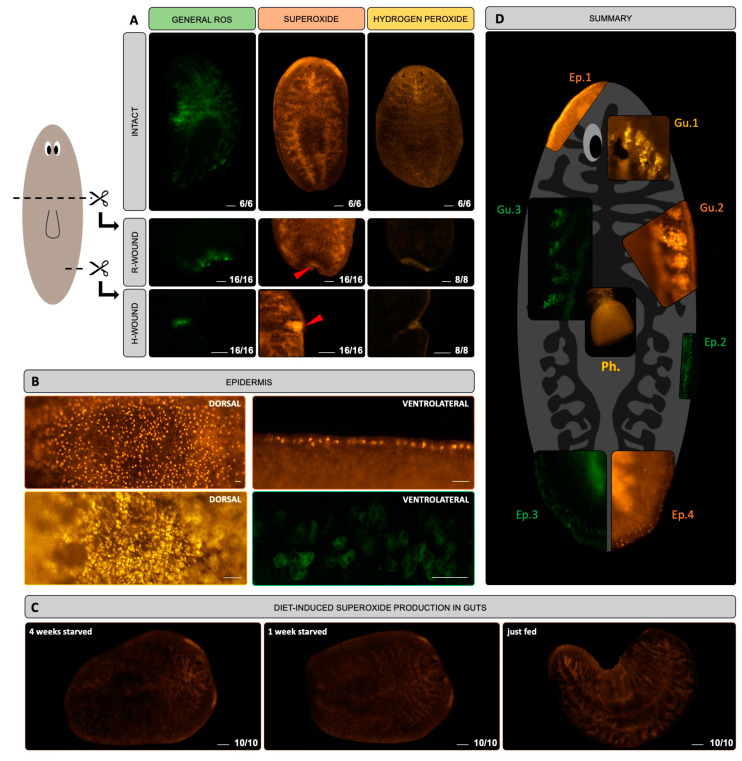
In vivo visualisation of reactive oxygen species (ROS) in general, and superoxide and hydrogen peroxide in specific. (**A**) Upper panel: intact animals stained for general ROS (green), superoxide (orange) and hydrogen peroxide (yellow). Middle panel: general ROS, superoxide and hydrogen peroxide production visualised in a regenerative wound (R-wound). Lower panel: general ROS, superoxide and hydrogen peroxide production visualised in a healing wound (H-wound). Scale bar represents 100 μm. (**B**) Detailed images of ROS-producing epidermal structures. Pictures in the left panels are taken at the dorsal side while pictures in the right panels are taken at the ventrolateral side. The colours of the panels correspond to the different stains (green: general ROS, orange: superoxide, yellow: hydrogen peroxide). Scale bar represents 20 μm. (**C**) Influence of diet on the production of superoxide in the guts. From left to right: intact animals starved for 4 weeks, intact animals starved for 1 week, intact animals that were fed just before the staining. Scale bar represents 100 μm. All pictures were taken with the same camera and software settings. (**D**) Summary figure containing images of the different in vivo stains: green (general ROS), orange (superoxide) and yellow (hydrogen peroxide), visualising epidermal structures (Ep.), structures in the guts (Gu.) and pharynx (Ph.).

Graphs were generated in R (version 3.5.3), Excel 2016 or GraphPad Prism (version 5.01, GraphPad Software, San Diego, CA, USA) and further modified (i.e., font enlargement, consistent colouring) in Adobe Illustrator 2020 (version 24.0.2, Adobe, San José, CA, USA) or Adobe Photoshop 2020 (version 21.2.3, Adobe). Microscopic pictures were processed to replace the background by a uniform background for better visualisation. Final manuscript figures were assembled in PowerPoint 2016 (Figure 1 and Figures S1–S4) or Adobe Illustrator (all other figures). The supplemental figures and corresponding captions can be found in Supplemental Material S3.

## 3. Results

In this study we aimed for a detailed characterisation of ROS and AOXs present in the freshwater planarian *S. mediterranea*, and further elucidated their role in the regeneration process. In our previous work, we visualised an amputation-induced ROS burst, using a general intracellular ROS stain [22]. We found that regeneration is impaired upon interfering with ROS production and that the presence of hydrogen peroxide (H_2_O_2_) plays a role in initiating the regeneration process [46]. The current study proceeds on these findings and focuses on both the pro-oxidative as the antioxidative side of the redox balance. Although often ignored, knowledge of all (counter)parts is of uttermost importance to fully conceptualise the function of the redox balance. First, we localised different types of ROS and the major antioxidant systems in the planarian body and studied their dynamics in both intact as well as regenerating animals. Second, after interfering with both sides of the redox balance, we assessed regenerative success and found that the severity and specificity of the phenotypic aberrations depended on the process that was manipulated. In the last part of this study, we focused on glutathione and glutathione-related processes, with a specific focus on stem cell responses.

### 3.1. ROS Are Detected in the Gut, at the Epidermis and in the Blastema of S. mediterranea

To study ROS production, we used three different fluorescent dyes, allowing us to discriminate between general ROS, superoxide and hydrogen peroxide. The major advantage of these stains is that they can be used in vivo, allowing us to capture fast-changing redox dynamics in different physiological conditions. In intact worms, a gut-like pattern is revealed by all staining methods (Figure 1A and Figures S1–S3). Part of the pharynx was also stained (Figure 1D—Ph.), although not in all observations. In the outer branches of the planarian intestine, multiple dot-like structures of 10 µm in size were observed (Figure 1D—Gu.1, Gu.2 and Gu.3, Figures S1–S3). The fluorescent intensity of the gut-like pattern was the most intense when worms were just fed, while the signal decreased after 1 or 4 weeks of starvation (Figure 1C).

In superoxide-stained worms, lateral signals were observed that were restricted to the head region and less intense anterior to the eyes (Figure 1A,D—Ep.1, Figure S2). At the dorsal side of the planarians, close to the surface and close to the epidermis, we observed superoxide and hydrogen peroxide positive structures, respectively 5 µm and 2,5 µm in size (Figure 1B, Figures S2 and S3), while at the ventrolateral side, general ROS and superoxide positive structures were stained, respectively 10 µm and 5 µm in size (Figure 1B,D—Ep.2, Ep.3, Ep.4, Figures S1 and S2).

Apart from studying ROS in intact worms, we also assessed ROS production during wound responses by inflicting healing and regenerating wounds. A healing response is triggered by injuries that require only wound healing (H-wounds), while regenerating wound responses involve tissue loss and require regeneration (R-wounds). We confirmed the general ROS production in R-wounds and H-wounds that we previously reported (Figure 1A) [22,46]. Additionally, the superoxide and hydrogen peroxide signals were also present in R-wounds and H-wounds, although not restricted to the wound itself (Figure 1A).

As planarians are known to exhibit autofluorescence, we included additional controls to discriminate between ROS-specific signals and autofluorescence (Figure S4A). We observed that the general characteristics and spatial patterning of the autofluorescent signals were different from the signals obtained after ROS staining. Together with the observation that the fluorescent intensity after hydrogen peroxide and superoxide staining was respectively 5.75 and 6.86 times higher than the autofluorescence signal, we were able to discriminate between autofluorescence and ROS specific signals. Secondly, ROS specificity was confirmed by means of exposure to the ROS inhibitor DPI (Figure S4B).

### 3.2. Antioxidative Genes Are Expressed in the Planarian Gut, Stem Cells and Epidermal Lineages

**Figure 2:**
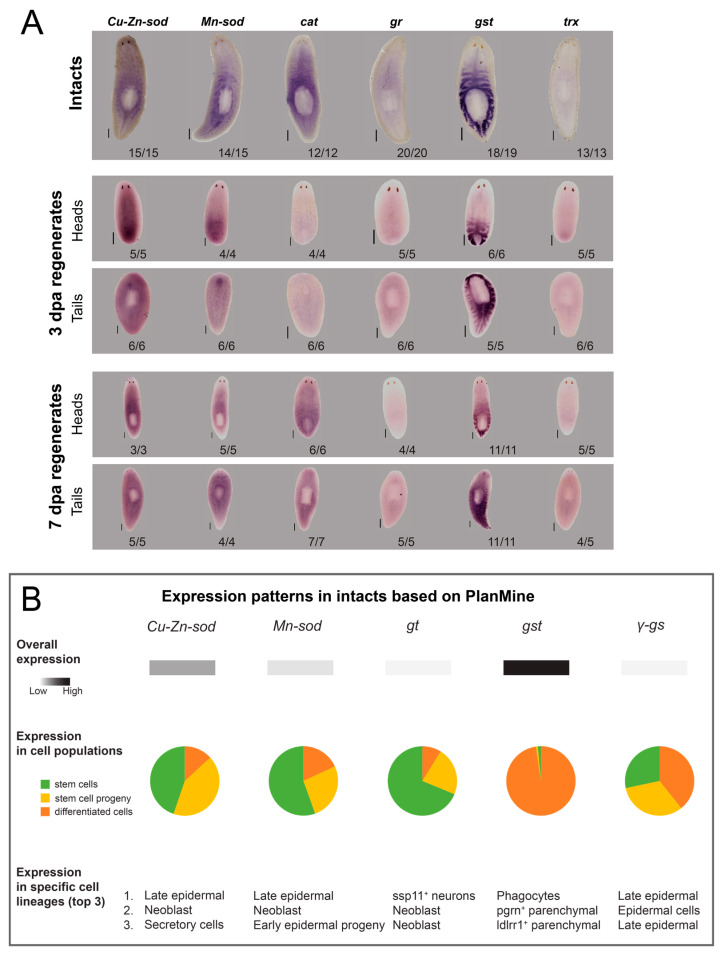
Localisation of major antioxidant systems in the planarian body. (**A**) Colorimetric in situ hybridizations of *Cu-Zn-sod*, *Mn-sod*, *cat*, *gr*, *gst* and *trx* in intact planarians and regenerating heads and tails 3 and 7 dpa. Scale bar represents 200 µm. (**B**) Expression levels of *Cu-Zn-sod*, *Mn-sod*, *gr*, *gst* and *γ-gs* in intact worms, based on transcriptome data deposited in PlanMine v3.0. (MPI-CBG, Dresden, Germany) (*sod*: superoxide dismutase, *cat*: catalase, *gr*: glutathione reductase, *gst*: glutathione-S-transferase, *γ-gs*: γ-glutamylcysteine synthetase, *trx*: thioredoxin, dpa: days post amputation).

In addition, we investigated the location and dynamics of antioxidative genes. In both intact and regenerating worms, we visualised the expression of six different antioxidative genes (*Cu-Zn-sod*, *Mn-sod*, *cat, gr*, *gst* and *trx*) (Figure 2A). *Gst* (*glutathione-S-transferase*) expression was the highest, followed by *cat*, *Cu-Zn-sod* and *Mn-sod*, while the expression of *gr* and *trx* was the weakest. For *gst* and *cat*, a gut-like pattern was observed, although the pharynx was not stained. The expression of *Cu-Zn-sod*, on the other hand, was clustered around the pharynx, possibly located in the secretory cells and/or stem cells. Similarly, the expression of *Mn-sod*, *gr* and *trx* was predominantly found in the cells surrounding the gut in a stem cell-like pattern.

A comparison with publicly available expression data of the PlanMine database allowed us to confirm the expression patterns of *Cu-Zn-sod*, *Mn-sod*, *gr* and *gst* (Figure 2B). *Cu-Zn-sod* and *Mn-sod* were mainly expressed in stem cells or stem cell progeny and, more specifically, in late epidermal and neoblast lineages, while *gr* was found in neurons and neoblast lineages. In contrast, *gst* expression was predominantly found in differentiated cells such as phagocytes and parenchymal cells (Figure 2B—expression in cell populations and specific cell lineages). The expression of *γ-gs* was approximately equally expressed in stem cells, stem cell progeny (i.e., late epidermal lineages) and differentiated epidermal cells (Figure 2B—expression in cell populations and specific cell lineages).

### 3.3. Transcripts of Antioxidative Genes Show Temporal Fluctuations during the Regeneration Process

To understand the spatiotemporal dynamics of redox molecules, we additionally studied the expression of antioxidative enzymes during the regeneration process. As we observed ROS at the wound site in the early phases of regeneration (Figure 1 and Pirotte et al. 2015 [22]), we followed antioxidant gene expression in the first 24 h of regeneration in the blastemas (Figure S5A). For *Cu-Zn-sod* and *Mn-sod*, we found a consistent net increase in the first 24 h. Other genes (*cat*, *gr*, *gst*) were also included; however, their expression patterns were more variable between the different experimental replicates.

The net increase in antioxidative gene expression was also reflected in previous studies, which we consulted via the publicly available gene expression profiles in PlanMine. More specifically, in regenerating head fragments, a net increase in the expression of *Cu-Zn-sod*, *Mn-sod*, *gr*, *gst* and *γ-gs* during the first 36 h of the regeneration process was reported (Figure S5B). This increase continues after 36 h for *Cu-Zn-sod*, *Mn-sod* and *gst*, while for *gr* and *γ-gs* a plateau was reached. In tail fragments, a strong increase in the expression of *Cu-Zn-sod*, *Mn-sod* and *gst* was observed 36 h after amputation, while the increase in expression of *gr* and *γ-gs* was less pronounced.

### 3.4. Exposure to Redox-Interacting Compounds Results in Impaired Regeneration

**Figure 3:**
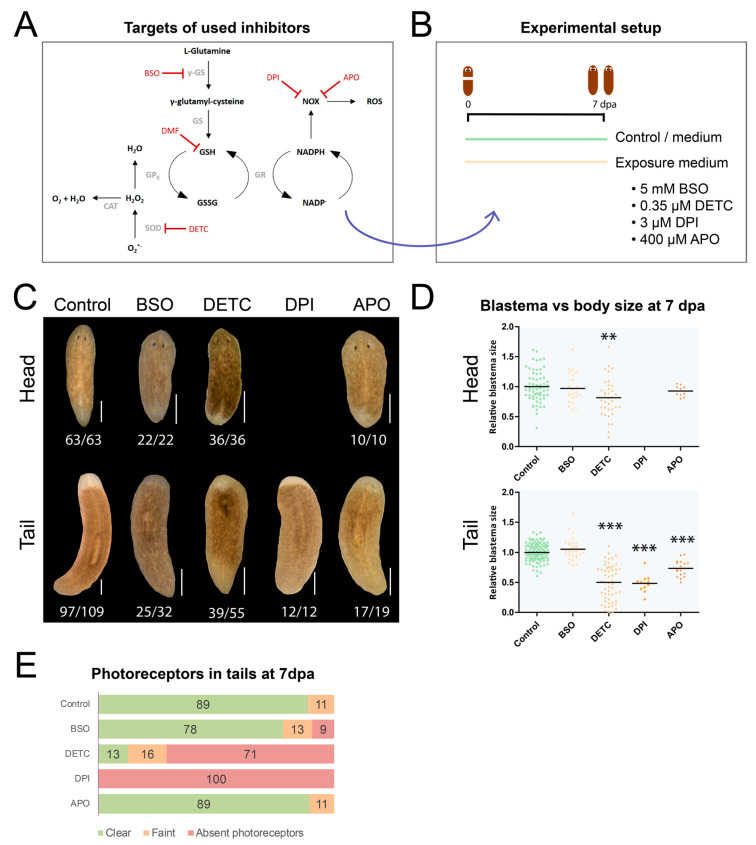
Phenotypes after interfering with the redox balance by exposure to redox influencing compounds. (**A**) Schematic overview of the studied pathways and the used inhibitors with their intended targets. Their respective off-target effects are listed in Table 1. (**B**) Overview of the experimental setup: worms were amputated in head or tail fragments and then exposed for 7 days to 5 mM BSO, 0.35 µM DETC, 3 µM DPI or 400 µM APO. (**C**) Phenotypes 7 dpa after exposure to redox-influencing compounds. The numbers below the worms represent how many times the depicted phenotype is observed versus the total number of observations. Scale bar represents 500 µm. (**D**) Blastema sizes normalised to the body size in head and tail fragments, relative to the control, 7 dpa (all compared with control: ** *p* < 0.01, *** *p* < 0.001, black line represents average per condition). (**E**) Percentages of clear, faint or absent photoreceptors in 7 dpa tails. (BSO: buthionine sulphoximine, DMF: dimethyl fumarate, DETC: di-ethyl dithio-carbamate, DPI: diphenyleneiodonium chloride, APO: apocynin, SOD: superoxide dismutase, CAT: catalase, GP_x_: GR: glutathione reductase, γ-GS: γ-glutamylcysteine synthetase, GS: glutathione synthetase, NOX: NADPH oxidase, ROS: reactive oxygen species, dpa: days post amputation.).

To study the functional role of redox molecules during the regeneration process, we induced regeneration and interfered with the redox balance by exposing head and tail fragments to four different compounds (Table 1, Figure 3A). Regenerative success was assessed after 7 days (Figure 3B). To study the consequences of inhibiting ROS production, we used DPI and APO, respectively; a nonspecific flavoprotein inhibitor; and NOX inhibitor (Figure 3A). Blastema sizes of tail fragments exposed to 3 µM DPI were significantly reduced (51%, *p* < 0.001) in size (Figure 3C,D) compared with control tails, and none of the DPI-exposed tails developed eyes (Figure 3E). Exposure to 400 µM APO did not significantly alter the phenotype and blastema size of head fragments, while in tail fragments the blastema was 26% smaller compared with control tails (*p* < 0.001) (Figure 3C,D). The majority (89%) of the tail fragments developed clear eyes, while faint eyes were observed in 11% of the fragments (Figure 3E).

Apart from interfering with the pro-oxidative side of the redox balance, we also interfered with antioxidant activity. An exposure to 0.35 µM DETC inhibited SOD activity for approximately 60% (*p* < 0.05) (Figure S6F) and induced a more severe phenotype. Head fragments exposed to 0.35 µM DETC showed a slightly reduced blastema size (19% smaller compared with control, *p* < 0.01) (Figure 3C,D). In tail fragments, a 50% reduction in blastema size was observed (*p* < 0.001) (Figure 3D), accompanied by 71% of the tail fragments developing without eyes and 16% with faint eyes (Figure 3E). In comparison with the pharmacological inhibition by DETC, interfering with SODs at the gene expression level via RNA interference resulted in a less severe phenotype, only affecting eye development to a lesser extent (Figure S6A). The blastema sizes were not affected after 7 days of regeneration (Figure S6B), although in developing tails with reduced *Cu-Zn-sod* gene expression, 11% of the photoreceptors were faint (Figure S6E). Additionally, in the dKD (double knockdown) condition, 11% of the photoreceptors were faint and 17% were absent. Control worms and worms with a reduced *Mn-sod* expression all developed clear eyes. Regarding the number of proliferating stem cells between the different conditions, no significant differences were observed (Figure S6C,D). The KD effect of both genes was confirmed (Figure S6G).

In addition, a 7-day exposure to 5 mM BSO, a compound that blocks the initial and rate-limiting step in glutathione synthesis by γ-GS (Figure 3A), resulting in approximately 50% (*p* < 0.001) reduction in glutathione content (Figure S7A), did not significantly alter the blastema sizes of head and tail fragments compared with control worms (Figure 3C,D). In 78% of the tail fragments, eye development was normal and resulted in two clear photoreceptors after 7 days of development (Figure 3E). However, in 13% of cases, faint eyes were observed, and 9% of the tail fragments did not develop eyes.

### 3.5. An In-Depth Study of Glutathione, Its Location and Function

Due to the interesting patterns in glutathione-related genes, we characterised this metabolite in more detail by determining and interfering with the total glutathione (GSH) levels in different experimental set-ups. To determine if GSH is located in the mucus layer, we removed the mucus layer via a treatment with NAC. To verify if GSH is mainly present in stem cells, as in other animals, we depleted the stem cell population by lethal radiation or via *H2B* KD [47]. All three setups did not result in decreased GSH levels (Figure S5C), suggesting that GSH is present in other structures and cell-types. We further characterised how glutathione levels evolve during regeneration and found that GSH was significantly (*p* < 0.001) increased at the very beginning (0 and 30 mpa) of the process compared with later stages (1 hpa, 3 hpa, 1 dpa and 3 dpa) (Figure S5D).

**Figure 4:**
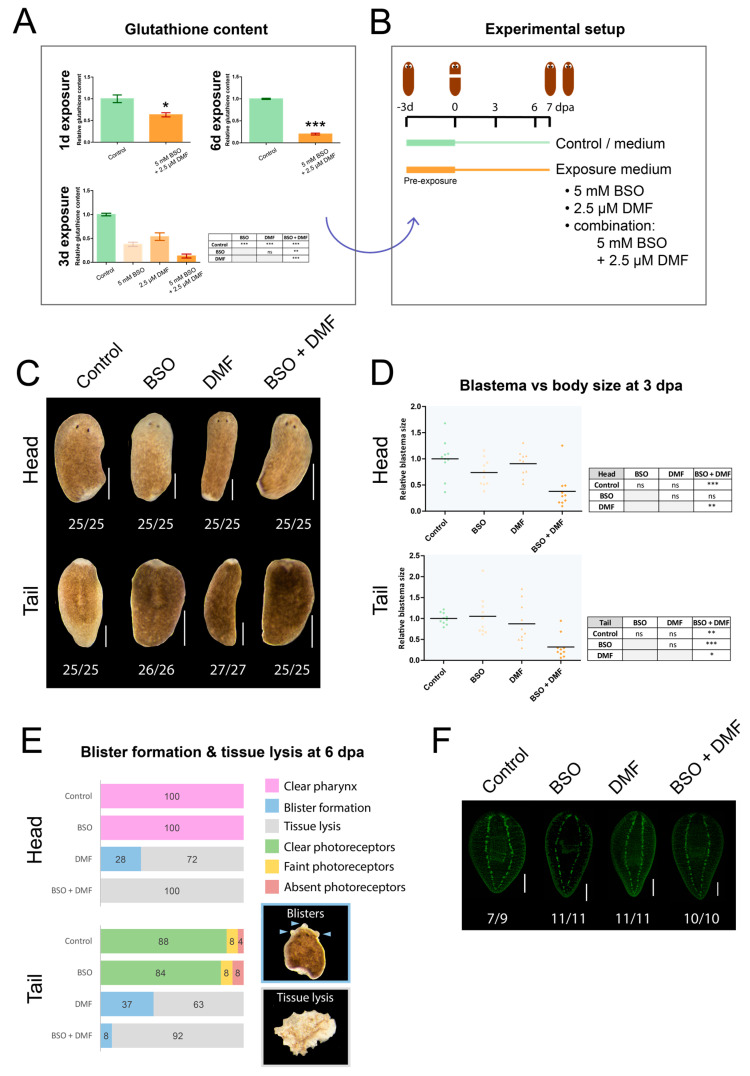
Glutathione content and phenotypes after interfering with the redox balance by exposure to redox-influencing compounds. (**A**) Average glutathione content in intact animals after exposure to 5 mM BSO and 2.5 µM DMF for 1, 3 or 6 days (*n* = 4, * *p* < 0.05, ** *p* < 0.05, *** *p* < 0.001, error bars represent standard error). (**B**) Overview of the experimental setup for panel C–E: worms were pre-exposed for 3 days to 5 mM BSO, 2.5 µM DMF or a combination of both. Next, they were amputated in head or tail fragments and allowed to regenerate in the same exposure solutions. (**C**) Phenotypes 3 dpa after exposure to glutathione interfering compounds. The numbers below the worms represent how many times the depicted phenotype is observed versus the total number of observations. Scale bar represents 500 µm. (**D**) Blastema sizes normalised to the body size in head and tail fragments, relative to the control, 3 dpa (ns: not significant, * *p* < 0.05, ** *p* < 0.01, *** *p* < 0.001, black line represents average per condition). (**E**) Percentages of worms that form blisters and undergo tissue lesions in 6 dpa head and tail fragments. (**F**) Neurodevelopment in 5 dpa tail fragments, stained by anti-synapsin. Scale bar represents 200 µm. The numbers below the worms represent how many times the depicted phenotype was observed versus the total number of observations. (BSO: buthionine sulphoximine, DMF: dimethyl fumarate, dpa: days post amputation).

In order to further elucidate the functional role of glutathione in regeneration, we depleted the glutathione content by pharmacological inhibition and determined the resulting phenotypes. We found that prolonged exposure to a combination of BSO and DMF was needed (Figure 4A and Figure S7A), suggesting that planarians contain high levels of this antioxidative metabolite. While shorter exposure times and/or lower concentrations resulted in ±63–44% glutathione content (Figure 4A and Figure S7A), a combined exposure to 2.5 µM DMF and 5 mM BSO for 3 or 6 days resulted in the lowest levels of glutathione (approximately 15%, *p* < 0.001) (Figure 4A). The phenotype and behaviour of intact animals was not affected by moderate or severe glutathione reduction (not shown). However, a 3-day exposure to 5 mM BSO and 2.5 µM DMF, followed by amputation and regeneration in the same exposure solutions (Figure 4B), resulted in regenerative aberrations. After 3 days of regeneration, aberrant phenotypes were observed (Figure 4C): a significant reduction in blastema size was observed in heads exposed to a combination of BSO and DMF (62% compared with control: *p* < 0.001, 53% compared with DMF: *p* < 0.01) (Figure 4D). In 3 dpa tails, combined exposure of BSO and DMF resulted in significantly smaller blastemas in developing tails (68% compared with control: *p* < 0.01, 73% compared with BSO: *p* < 0.001, 55% compared with DMF: *p* < 0.05). At later stages in the regeneration process, all BSO-exposed heads developed normally, similar to the control condition. However, in 6 dpa tails, a fraction developed faint and no photoreceptors (both 8%). When DMF was applied in a single exposure set-up, we observed impaired mobility and sidewise lying, which could not be reversed to normal by the worm (not shown). This was accompanied by severe tissue lesions in 72% of the 6 dpa heads and 63% of the 6 dpa tails (Figure 4E). The remaining heads and tails showed blister formation and lysed in the following days. The combined exposure of BSO and DMF resulted in tissue lysis in 100% of 6 dpa heads and 92% of 6 dpa tails. In addition, 5 dpa exposed tails showed a trend towards delayed brain development compared with the control condition (Figure 4F).

**Figure 5:**
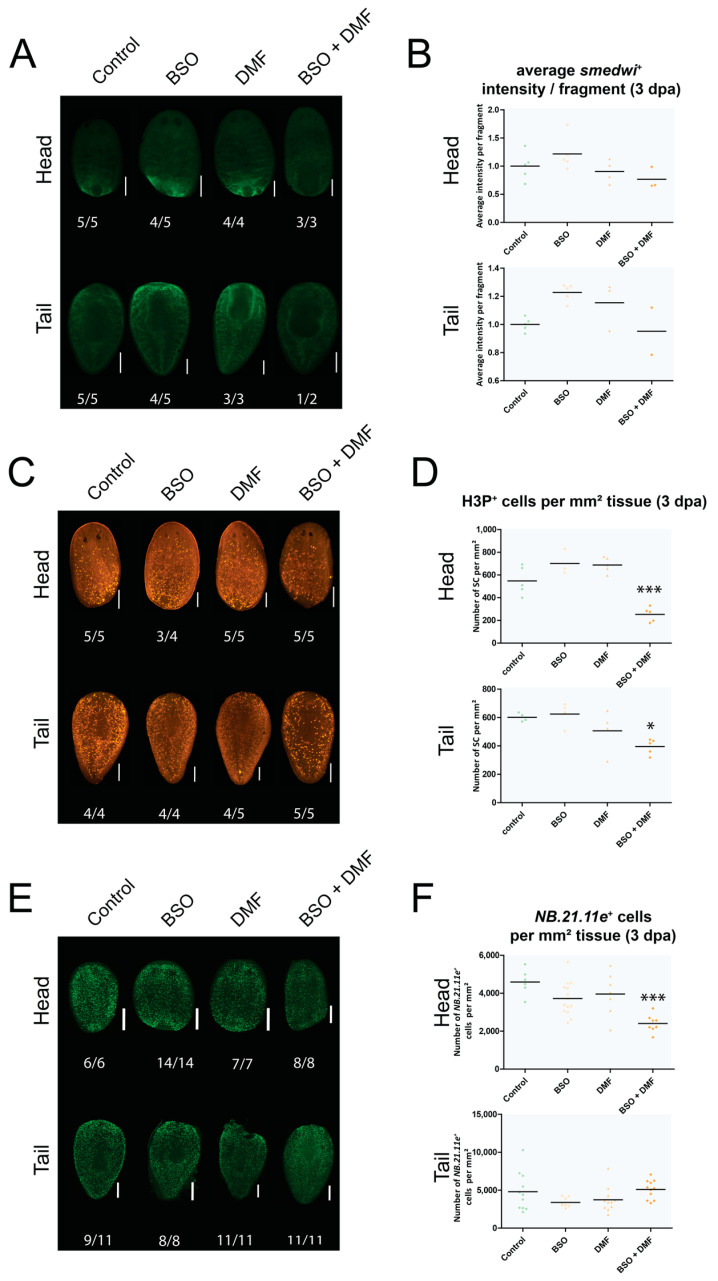
Stem cell responses in 3 dpa head and tail fragments after exposure to compounds that interfere with glutathione content (5 mM BSO, 2.5 µM DMF, pre-exposed for 3 days). (**A**) *Smedwi-1* stain as a measure for the total number of stem cells in 3 dpa head and tail fragments. The numbers below the fragments represent how many times the depicted pattern is observed versus the total number of observations. Scale bar represents 500 µm. (**B**) Average intensity of *smedwi*^+^ signal relative compared with control, 3 dpa (all ns, black line represents average per condition). (**C**) Histon H3P stain as a measure for the number of proliferating stem cells in 3 dpa head and tail fragments. The numbers below the fragments represent how many times the depicted pattern is observed versus the total number of observations. Scale bar represents 500 µm. (**D**) Number of histon H3P^+^ cells, normalised over the surface area of the head or tail fragments, 3 dpa (all compared with control: * *p* < 0.05, *** *p* < 0.001, black line represents average per condition). (**E**) *NB.21.11e* stain as measure for the number of early progeny cells per mm^2^ tissue in head and tail fragments, 3 dpa. Scale bar represents 200 µm. (**F**) Number of *NB.21.11e*^+^ cells per mm^2^ tissue in head and tail fragments, 3 dpa (compared with control: *** *p* < 0.001, black line represents average per condition). (BSO: buthionine sulphoximine, DMF: dimethyl fumarate, dpa: days post amputation, ns: not significant, SC: stem cells).

In addition, we studied stem cell responses (i.e., number of stem cells present, number of proliferating or differentiating stem cells) underlying these phenotypes. Therefore, we determined the average fluorescence intensity of the *smedwi*^+^ signal, which is a proxy for the number of stem cells present. No significant difference was detected between the different exposure conditions (i.e., single exposure to BSO, single exposure to DMF, combined exposure to BSO and DMF) at 3 dpa (Figure 5A,B). However, the number of proliferating stem cells (H3P^+^) was significantly reduced in the 3 dpa head (*p* < 0.001) and tail (*p* < 0.05) fragments that were exposed to a combination of BSO and DMF, in comparison with the corresponding control conditions (Figure 5C,D). Fragments exposed to BSO alone or DMF alone did not significantly differ from their controls. In addition, we found that a combined exposure of BSO and DMF significantly reduced (*p* < 0.001) the number of *NB.21.11e*^+^ cells, which are a measure for early progeny, in 3 dpa heads (Figure 5E,F).

Worms exposed to the lowest concentration (1.25 µm) of DMF in combination with 5 mM BSO showed no alterations in (proliferating) stem cells compared with controls (Figure S8B,C). However, in 7 dpa tails, DMF exposure led to significantly less (*p* < 0.01) stem cells compared with the control (Figure S8B). In addition, in 3 dpa heads, DMF exposure resulted in a significant increase (*p* < 0.05) in proliferating stem cells (Figure S8C). Worms exposed to a combination of 5 mM BSO and 2 µM DMF showed an increased number of stem cells in 3 dpa heads (*p* < 0.05) and tails (*p* < 0.01) (Figure S8B). The number of proliferating stem cells was also significantly increased (*p* < 0.01) in 3 dpa heads compared with controls (Figure S8C). A short pre-exposure (24 h) to 5 mM BSO, combined with 2.5 µM DMF and regeneration in the same exposure solution, resulted in a significant decrease in the amount of stem cells in 3 dpa tails (*p* < 0.05) (Figure S8B). The number of proliferating stem cells was also significantly lower (*p* < 0.01) compared with control tails (Figure S8C).

Apart from pharmacological inhibition, we also interfered with expression of the *gst* gene, which is related to glutathione function. Compared with water-injected controls, a reduction of 95% (*p* < 0.01) in *gst* gene expression was observed (Figure S7E). The reduction resulted in mild phenotypes after 7 days of development (Figure S7B), especially linked to photoreceptor development (Figure S7C). Control tails showed clear photoreceptors in 97% of the cases, while in 3% of the cases they were faint. In the *gst* KD condition, 7% showed faint photoreceptors, while 10% had absent photoreceptors. Neurodevelopment was normal for 7 dpa regenerating tails with reduced *gst* expression (Figure S7D). Both the number of proliferating stem cells (Figure S7F,G) and early progeny cells (Figure S7H,I) were not affected by the decrease in *gst* expression.

## 4. Discussion

Oxygen plays a central role in life. Not only is it indispensable for the daily functioning and physiology of many animals, oxygen has also shaped the evolution of life on Earth [7]. The permanent accumulation of oxygen in our atmosphere has allowed evolution towards an aerobic metabolism to take place, resulting in a more efficient energy production and the emergence of multicellular organisms. However, the toxic by-products, such as ROS, increased the demand for adequate oxygen sensing and protective systems. This drove the concurring evolution of oxygen sensors and antioxidant systems [7,48], illustrating the inseparable and dynamically coupled character of ROS and AOXs. Although this dynamic link is often overlooked, we believe that an integrated study of pro-oxidative and antioxidative actions is indispensable to fully grasp the complexity of redox regulation in animal physiology. Previous studies already show how this redox balance is involved in diverse physiological processes including development, tissue repair and regeneration [1,2]. Additionally, in planarians, ROS were shown to be involved in initiating regeneration [46] and appear to play a role in modulating stem cell differentiation [22]. However, tight regulation with AOXs remains unexplored so far. This exploratory study is the first to describe the spatiotemporal and functional profiles of different redox molecules in the planarian *S. mediterranea* in different physiological conditions.

Our data (Figure 1A,C and Figures S1–S3) clearly show that redox activity takes place in the gut, among others. The planarian intestine is directly connected with the exterior environment via the pharynx and consists of a single anterior branch and two posterior primary branches, each of which ramifies in smaller branches [49]. In humans and mice, intestinal ROS are produced in response to bacteria and regulate local wound healing [50]. This corresponds with our findings, as we detected superoxide and hydrogen peroxide positive signals (that did not show autofluorescence) in the smaller branches of the intestine. The exact identity of the structures still has to be elucidated, but we assume that these ROS are either associated with planarian gut cells, microorganisms or remnants of liver/food. Feeding increased the intensity of the fluorescent signal in the gut (Figure 1C) as also has been shown in other animals (byproduct in absorption of food), although we could not completely exclude signal aspecificity in this case. In other conditions, we were able to discriminate between autofluorescent and ROS-specific signals by means of different spatiotemporal patterns and the intensity of the ROS signal. In all live imaging settings, the ROS signal was strong enough to overcome autofluorescence (Figure S4A). Secondly, live imaging does not allow a controlled and equal uptake of the ROS detecting fluorophore in all cells and organs. Hence, we visualised the expression of some anti-oxidants, such as *gst* and *cat* and noticed a similar gut-related pattern (Figure 2A,B). Also the high expression of *gst* in the gut (Figure 2A), and more specifically in phagocytic cells (Figure 2B), is in line with its detoxifying function by conjugating glutathione to xenobiotics and suggests a link with the metabolisation of food and other particles in the gut. Phagocytes are one of the three cell types that have been described in the intestinal epithelium, apart from secretory goblet cells and basally located ‘outer’ intestinal cells, and allow the process of phagocytosis to occur [51,52], suggesting an involvement of glutathione in the innate immune system. The other enzymes (*Cu-Zn-sod*, *Mn-sod*, *gr* and *trx*) were less abundant (Figure 2A,B) and expressed in stem cells or in epidermal lineages (Figure 2B). The latter corresponds to the epidermal ROS signal in our measurements (Figure 1A,B and Figures S1–S3). From literature and planarian transcriptomics databases, we know that *Cu-Zn-sod* and *Mn-sod* are present in (differentiating) epidermal cells (Figure 2B), which again relates to the superoxide signal at the epidermis lateral of the head region (Figure 2A). As the epidermis forms a first barrier against the outside world and protects against invasion of pathogens and xenobiotics [51], it is possible that ROS are involved in this defence reaction. Another explanation might involve the cilia and rhabdites, which cover the epidermis and are probably sources of redox metabolism (Figure 1B and Figures S1–S3).

To obtain a deeper understanding of the dynamics and the physiological role of the redox molecules, we studied them over time and functionally interfered with these processes. As several of the enzymes were expressed in stem cells and/or showed stem cell-like expression patterns (i.e., *Cu-Zn-sod*, *Mn-sod*, *gr* and *trx*), we specifically focused on their role in the regeneration process. Our data confirmed previous findings on the presence of a ROS burst after a R-wound [22,46] and additionally showed that ROS are also generated in H-wounds (Figure 1A), which do not require regeneration or the formation of a blastema. Inhibition of this ROS burst by DPI or APO resulted in regeneration defects (Figure 3C,D), confirming the studies of Pirotte et al. 2015 [22] and Jaenen et al. 2021 [46]. The effect of DPI was more severe than that of APO, which we attribute to the less specific nature of DPI (Table 1). We hypothesise that the lack of photoreceptors is due to disturbances in the differentiation process, as DPI treatment resulted in more undifferentiated stem cells at the wound site of regenerating worms [22]. In addition, a decreased brain size and cephalic ganglia suggest defects in central nervous system regeneration, accompanied by a reduced number of mechanosensory and GABAergic neurons. Especially the transition from the undifferentiated to the differentiated state seems to be affected, with a pronounced effect in neuronal cells. Future research regarding the differentiation into photoreceptors might reveal additional insights.

Together with a ROS burst at the wound site in the initial phases of regeneration, a net increase in the expression of antioxidative genes *Cu-Zn-sod* and *Mn-sod* was observed in the blastemas in the beginning of the regeneration process (Figure S5A). At later time points, more specifically 3 dpa and 7 dpa, no expression was detected in the blastemas (Figure 2A). SOD prevents the accumulation of superoxide and is the only eukaryotic enzyme known to be able to do this [53]. Detoxification is achieved by converting it to hydrogen peroxide that, in its turn, is further converted to water by CAT [54]. An elevated concentration of superoxide can damage and inactivate proteins containing iron-sulfur clusters, with a loss of SOD function consequently leading to a decreased lifespan in yeast, flies and mice [53]. In our experiments, a 60% reduction of SOD activity, using the pharmacological inhibitor DETC, resulted in decreased blastema sizes in both developing head and tails (Figure 3C,D). This was accompanied by aberrant photoreceptor development as most were not developed 7 dpa (Figure 3E), which suggested defects in neurodevelopment. As often the case with enzymatic inhibitors, we cannot exclude possible off-target effects, as DETC has metal chelating properties and can interfere with other important enzymes such as matrix metalloproteinases, important in tissue remodelling [26,27,33]. Hence, we confirmed our results after transcriptional KD of *Cu-Zn-sod* and *Mn-sod* via RNA interference (Figure S6G). A transcriptional downregulation of *Cu-Zn-sod* resulted in a small percentage of delayed eye development, while *Mn-sod* KD tails all developed normally (Figure S6A,E). A compensatory mechanism is probably in place [55] as only the double KD resulted in more strongly delayed eye development (Figure S6A,E).

Due to its known role in tissue repair and regeneration in other animals, we additionally focused on glutathione and related enzymes. During the first phases of regeneration, an increase in glutathione content (Figure S5D) and in the expression of glutathione-related genes *γ-gs*, *gr* and *gst* was measured in head and tail fragments (Figure S5B), suggesting that the worms respond to the amputation-induced ROS burst. The tri-peptide glutathione exists in a reduced (GSH) and oxidised (GSSG) form, and the balance between both determines the redox state of the cell or organism, being hugely in favour of GSH in a normal physiology [56]. The study of Natarajan 2015 [57] showed that reduced GSH levels were almost 200 times higher than GSSG in planarians. Our results show a similar trend, as the measured GSSG levels were below the detection limit (not shown). Interfering with the first step in the synthesis of glutathione, by BSO-mediated blocking of *γ-GS* activity, only reduced glutathione content by half (Figure S7A) and resulted in only a third of the tails displaying delayed photoreceptor development (Figure 3C). Exposure to a combination of two different inhibitors for at least 3 days was needed to severely reduce the glutathione content (Figure 4A and Figure S7A), indicating a high concentration—or turnover—of available glutathione. The severe reduction had no consequences for intact animals; however, when amputated, glutathione depletion resulted in severe phenotypes, characterised by regenerative failure and tissue lesions (Figure 4C,E). Underlying, significantly less proliferating stem cells were detected in glutathione-depleted regenerating worms (Figure 5C,D), resulting in significantly smaller blastema sizes (Figure 4C,D). In head fragments, the number of early progeny cells was also significantly reduced (Figure 5E,F). Our preliminary findings suggest that glutathione is not (predominantly) present in stem cells (Figure S5C), and correspond to the previously published observation that the planarian blastema, a region without stem cells, was high in glutathione content [57]. Removing the epidermal mucus did not significantly decrease glutathione (Figure S5C), and we hypothesise that glutathione is present in other cell types, among which are gut cells (Figure 2). At the beginning of regeneration, which coincides with the ROS burst [22], high levels of glutathione were observed, followed by a decrease (Figure S5D). In the early stages of zebrafish development, glutathione was found to increase between the fertilisation and hatching stage, while after hatching, the redox potential was not altered [58,59]. In several other animal [60,61] and human studies [62,63], the concentration of glutathione declines with age, accompanied by a shift towards the pro-oxidative side of the balance [64], the latter being a hallmark of ageing [65]. Glutathione was shown to protect telomeres, with a peak in telomerase expression during the highest glutathione levels [66]. However, the exact function of the high glutathione levels in planarians remains to be determined.

Taking the data of the different experiments together indicates that interfering with both the antioxidative and pro-oxidative part of the redox balance results in similar phenotypes, affecting blastema sizes, stem cell dynamics and neurodevelopment (Figure 3, Figure 4 and Figure 5) [22]. In general, regenerating tail fragments were more severely affected than head fragments, which is possibly related to the complex task of fully regenerating functional cephalic ganglia and photoreceptors, while head fragments already have these and only need to regenerate less complex structures such as a pharynx and nerve strands.

Studying redox molecules is not straightforward and comes with several specific challenges. By combining multiple approaches, we were able to get a preliminary understanding of their location, dynamics and function. Via in vivo fluorescent staining and live imaging, we captured the fast-changing nature of ROS in different physiological stages of planarians. As autofluorescence is often present in anatomical tissues, we performed additional measurements to distinguish between autofluorescent and ROS-specific signals. To exclude a potential imbalance in the uptake ratios of the drug or ROS-detecting fluorophore in the different tissues, we complemented our findings with gene expression data of the respective antioxidative counterparts. We compensated possible off-target effects of enzymatic inhibitors (Table 1) by supplementing our findings with knockdown experiments that interfere with another level, namely the level of gene expression. Together, our approach of combining different methodologies takes a step forward in the characterisation of ROS and the antioxidant system in planarians. The next step is to unravel the deeper underlying molecular mechanisms and pathways and their regulation in the regeneration process.

## 5. Conclusions

In this exploratory study, we discriminated between different types of ROS (i.e., general ROS, hydrogen peroxide and superoxide) and determined that the gut and epidermis of *S. mediterranea* are places with high redox activity, possibly linked to an important role in defence against external stressors. By looking at the interplay between ROS and antioxidants, we observed that planarians have a well-equipped antioxidant system. Interfering with ROS production or SOD activity resulted in impaired regeneration, affecting regenerating tail fragments more than heads and was characterised by reduced blastema sizes and absent photoreceptors. Interfering with the highly abundant metabolite glutathione led to a decrease in proliferating stem cells and early progeny cells, resulting in tissue blisters and lesions. Together, these results show that we need to look at the interaction between the pro-oxidative and antioxidative side to fully explore the exact role of redox processes in planarian physiology and regeneration.

## Figures and Tables

**Table 1:** Inhibitors used in this study to interfere with the redox balance, their aimed targets and known off-target effects.

Inhibitor	Intended Target	Off-Target Effects	References
APO	Selectively inhibits NOX and acts on the translocation of the cytoplasmic subunits	Unknown	Wind et al. 2001 [24], Stefanska and Pawliczak 2008 [25]
BSO	Inhibits γ-GS, the rate-limiting step in the production of glutathione	Inhibits trypanothione synthetase (important for protozoa)	Singhal et al. 1987 [28], Piwien-Pilipuk and Galigniana 2000 [31], Vazquez et al. 2007 [32]
DETC	Known inhibitor of superoxide dismutase by chelating metals	- Inhibits metalloproteinases - Interferes with NF_κ_B - Activates P38, Akt and ERK1/2	Takeuchi et al. 1996 [26], Dumay et al. 2006 [27], Liu et al. 2013 [33]
DMF	Depletes glutathione from the pool	- Activates GR - Activates NRF2	Dethlefsen, Lehman 1988 [29], Hoffmann et al. 2017 [34]
DPI	Non-specific flavoprotein inhibitor (incl. NOX), interferes with many different electron transporters	- Interferes with quinone oxidoreductase, cytochrome P450 reductase, nitric oxide synthetase, xanthine oxidase - Activates p53	Bedard and Krause 2007 [23], Wind et al. 2001 [24], Park et al. 2007 [35], Li and Trush 1998 [36]

APO: apocynin, BSO: buthionine sulphoximine, DETC: di-ethyl dithio-carbamate, DMF: dimethyl fumarate, DPI: diphenyleneiodonium, NOX: NADPH oxidase, γ-GS: γ-glutamylcysteine synthetase, GR: glutathione reductase.

## Data Availability

The data presented in this study are available from the authors.

## Supplementary Materials

The following are available online at https://www.mdpi.com/article/10.3390/biom11050714/s1. Excel file S1: Summary of database search (PlanMine 3.0), Table S1: Sequences of qPCR primers, Table S2: Sequences of primers used to generate RNAi probes, Table S3: Sequences of primers used to generate in situ probes, Figure S1: In vivo visualisation of reactive oxygen species (ROS) in general (additional images), Figure S2: In vivo visualisation of superoxide (additional images), Figure S3: In vivo visualisatoin of hydrogen peroxide (additional images), Figure S4: The fluorescent signal observed is ROS production specific and not related to autofluorescence and ROS production is inhibited after treatment with DPI, Figure S5: Localisation of major antioxidant systems in the planarian body (continued), Figure S6: Phenotypes after interfering with the expression of *Cu-Zn-sod* and *Mn-sod*, Figure S7: Glutathione content after exposure to inhibitors and phenotypes and stem cell responses after interfering with the expression of *gst*, Figure S8: Stem cell responses in regenerating worms after exposure to BSO and DMF in different concentrations.
