# Supplementary material for: A Spatiotemporal Characterisation of Redox Molecules in Planarians, with a Focus on the Role of Glutathione during Regeneration
Source: Biomolecules. 2021 May 11;11(5):714. doi: 10.3390/biom11050714 (PMC8150688; doi:10.3390/biom11050714)
Supplement: Supplementary file 1 [file biomolecules-11-00714-s001.zip › S2 Primer sequences.pdf]

Supplemental files

S2 Primer sequences

Table S1 –Sequences of qPCR primers

Gene	Forward primer (5’-3’)	Reverse primer (5’-3’)
gapdh	GCAAAACATTATTCCGGCTTC	GCACTGGAACTCTAAAGGCCA
β-actin	AGAACAGCTTCAGCCTCGTCA	ITGGAATAGTGCTTCTGGGCAT
rpl13	AGGTGTCCCAGCTCCTTATGA	GGCCCAATTGACAGAATTTTC
gm2ap	CCGTCAGATTAAAGCTCGGTT	TTTCGGACATTCGTTACCCAT
gst	CACCAGAAGAGAAGAAAGAAGAAC	TTTTCTCCAGCGAAAAATCC
Cu-Zn-sod	TTCATGCTGTATGCGTTTTG	AACCGTGTTTACCAGGAGTTAGA
Mn-sod	TGGGCTTGGTTAGGGTTGAA	AACGTCAAATCCTAGCAACGG

Table S2 –Sequences of primers used to generate RNAi probes

Gene	Forward primer (+ T7)	Reverse primer (+ T7)
gst	GAGCTGAACTGATTCGACTGG	TGTCATCTTCTCCCATGAACC
Cu-Zn-sod	AGTTCATGCTGTATGCGTTTTG	GCTAGTTTCGTGGCCTCCTT
Mn-sod	GCAATGCAAAATGCAATAAGA	CCATGTGCAATCTCCAAAA
H2B	AAGATCGCTGCTAAGTCTGTT	CGTGTTTAGCCAATTCTCCT

Table S3 –Sequences of primers used to generate *in situ* probes

Gene	Forward primer	Reverse primer (+ T7)
gst	GGCCAGAAGTGAAACCAAC	GGACGAGTATTAACCCATTCTTC
gr	TCGGATTAGCTGAGGAGGAC	CGATCAAAGCACACACGAAG
Cu-Zn-sod	ACGAATTTGGCGACAATACAA	CCCACACGCTAATCGACCAC
Mn-sod	GCACTGGAGCATCAATATC	CAAATCCTAGCAACGGCTTC
cat	CCCACACCTTCAAATTGGTC	GGTCAACGTCGCCAGTAAG
trx	CCGATAGCGACCTGGATG	GCTGCTCAAAATGATTGCTC
smedwi-1	GTGACGCAGAGAAACGGAAG	TTGGATTAGCCCCATCTTTG
NB21.11e	GTGATTGCGTTCGCGTATATT	ATTTATCCAGCGCGTCATATTC
