# Supplementary material for: A Spatiotemporal Characterisation of Redox Molecules in Planarians, with a Focus on the Role of Glutathione during Regeneration
Source: Biomolecules. 2021 May 11;11(5):714. doi: 10.3390/biom11050714 (PMC8150688; doi:10.3390/biom11050714)
Supplement: Supplementary file 1 [file biomolecules-11-00714-s001.zip › S3 Supplemental figures.pdf]

Supplemental files

S3 Supplemental figures

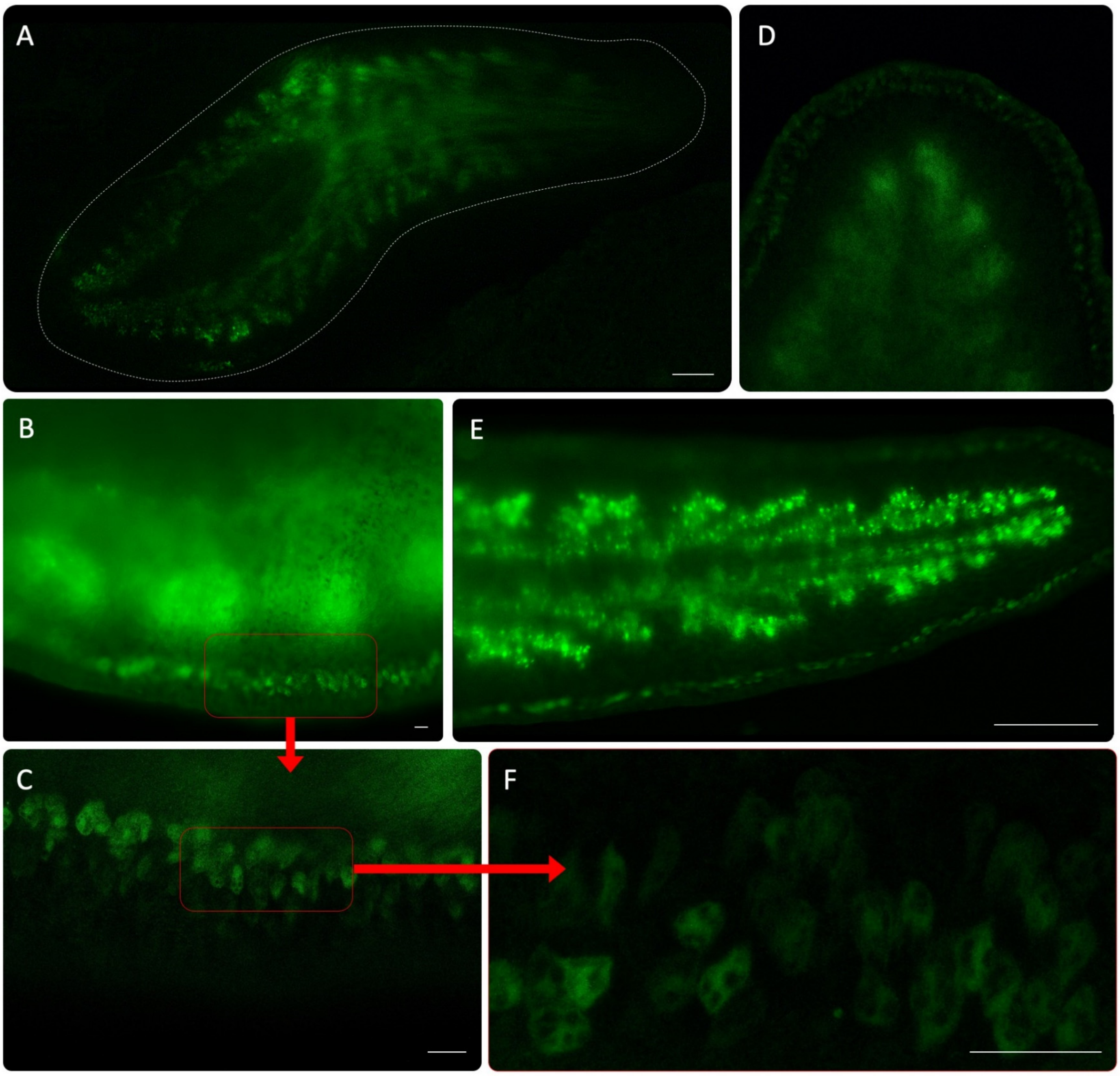

Figure S1 - *In vivo* visualisation of reactive oxygen species (ROS) in general (additional images). A Intact animal stained for ROS, imaged at the ventral site but focused slightly deeper at the height of the intestines, visualising a clear signal in the intestines. White dotted line indicates the border of the animal. Scale bar 100 µm. **B** Image of ROS-producing epidermal structures at the ventrolateral site of the animal. Red square is magnified in panel C. Scale bar 20 µm. **C** Magnified part of the image in panel B, visualising a clear signal in a specific type of epidermal cells laying in different undefined layers. Scale bar 20 µm. **D** Zoomed in image focused on the head region. Picture taken at the ventral site. Scale bar 20 µm. **E** Image of a clear signal observed in the intestines, tail region. Scale bar 100 µm. **F** Detailed image of ROS-producing epidermal cells at the ventrolateral site of the animal. This image is a magnification of the red square observed in panel C. Scale bar 20 µm.

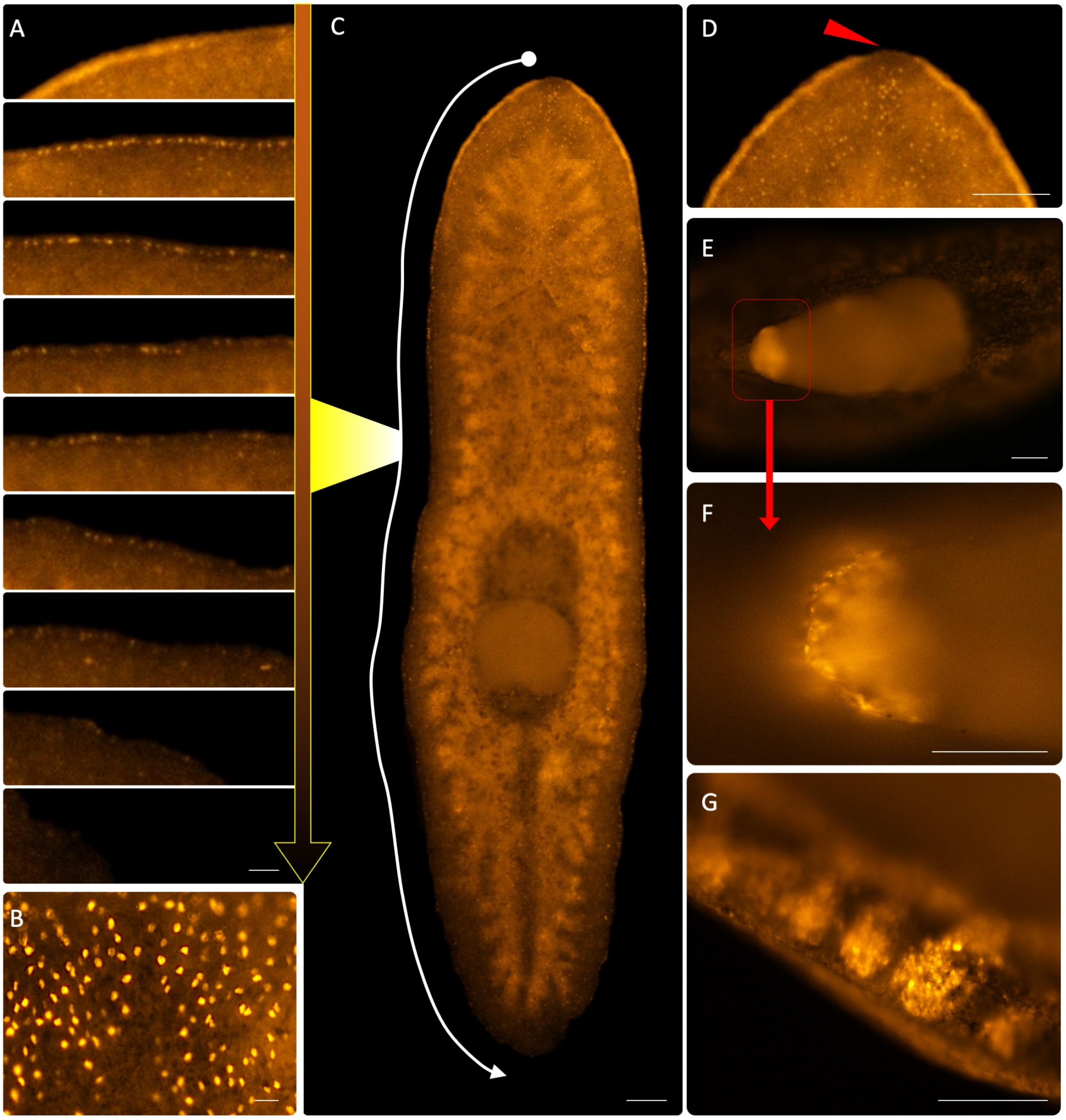

Figure S2 - *In vivo* visualisation of superoxide (additional images). **A** Detailed images of ROS-producing structures located in the ventrolateral epidermis cells, chronologically from top to bottom according the anterior posterior axis. This is again indicated by the arrow between panel A and C. Scale bar 20 µm. **B** Detail images of ROS-producing epidermal structures at the dorsal site of the animal. Scale bar 20 µm. **C** Intact animal stained for superoxide, imaged at the ventral site. The white arrow indicates the direction of the images presented in panel A. Scale bar 100 µm. **D** Zoomed in image of panel C, focused on the head region. Picture taken at the ventral site. Red arrowhead indicating the absence of signal at the most anterior tip of the animal while a clear signal is visible at the rest of the epidermis in the head region. Scale bar 100 µm. **E** Image of the pharynx. Red box indicating a clear signal at the attachment structure of the pharynx to the animal. **F** Zoomed in image of panel E. Both E and F, scale bar 100 µm. **G** Detailed image of a clear signal observed in the guts. Image visualises a gut branch with different intestinal lobes. Scale bar 100 µm.

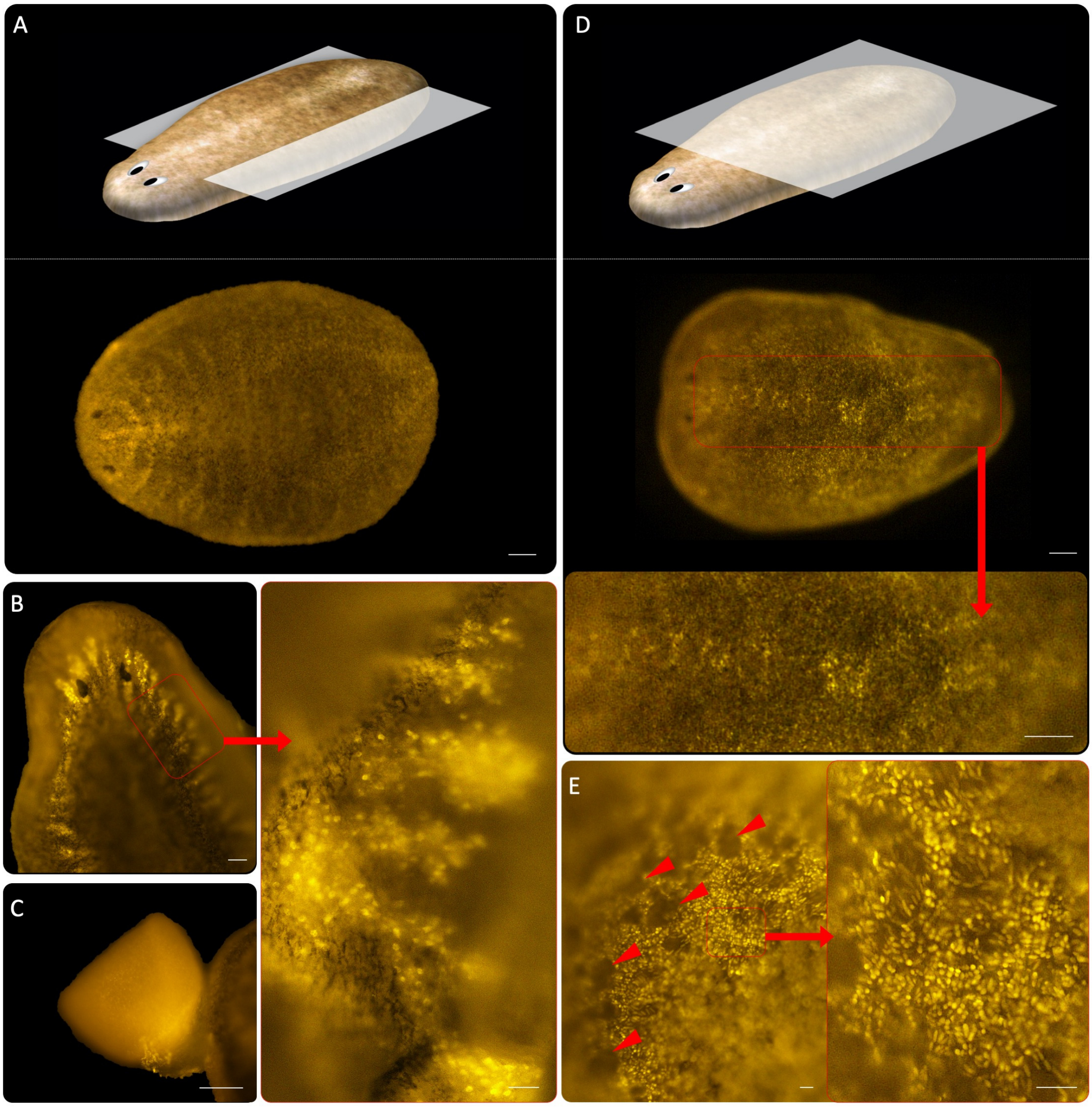

Figure S3 - *In vivo* visualisation of hydrogen peroxide (additional images). A Intact animal stained for superoxide, imaged at the ventral site but focused slightly deeper at the height of the intestines (visualised in the model at the top of the panel). Scale bar 100 µm. **B** Detailed image of a clear signal observed in the intestines, head region. Scale bar 100 µm. Red square is magnified in the panel at the right. Image visualises a gut branch with different intestinal lobes. A clear signal is observed in round, dot-like structures inside the intestines. Scale bar 20 µm. **C** Image of the pharynx. Scale bar 100 µm. **D** Intact animal stained for superoxide, imaged at the ventral site and focused at the most outer layer of the animal at the height of the epidermis (visualised in the model at the top of the panel). Red square is magnified at the panel below, indicating the presence of stripe over the anterior-posterior axis, observed as a brighter signal in the individual epidermal cells. Scale bar 100 µm. **E** Detailed images of the signal in epidermal cells at the dorsal site of the animal. Red arrowheads indicating the round spots on the dorsal site with less signal attributed to superoxide. The red square is again magnified in the panel at the right. Scale bar 20µm.

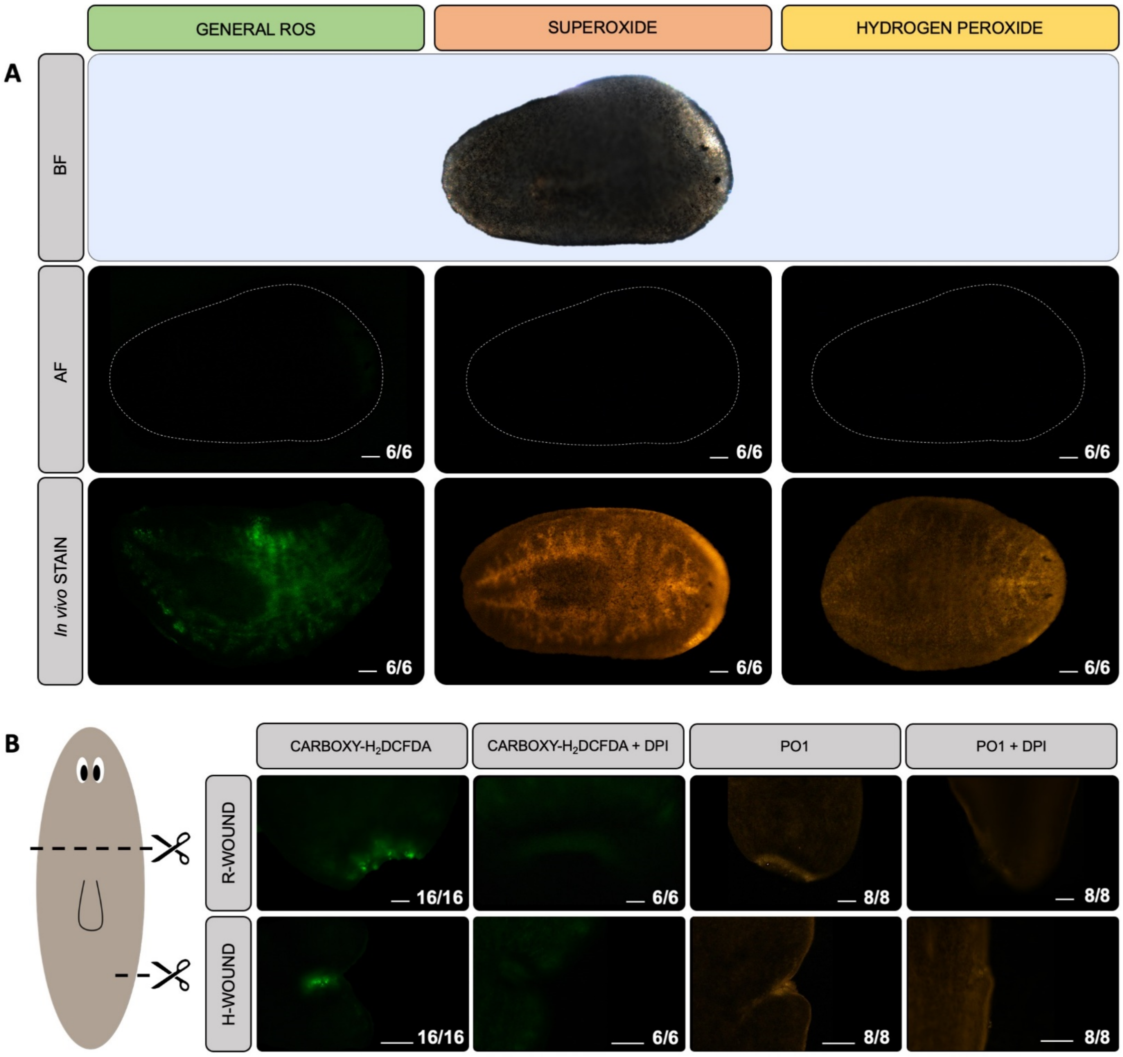

Figure S4 - The fluorescent signal observed is ROS production specific and not related to autofluorescence and ROS production is inhibited after treatment with DPI. A In the upper panel an intact animal is visualised under brightfield (BF). In the panels in the middle, autofluorescence (AF) for each individual stain is checked at the corresponding wavelength. The lower panels show the ROS stain specific signal in intact animals, as shown in Figure 1. Scale bar represents 100 µm. **B** The amputation setup for healing (H) and regenerative (R) wounds is displayed at the left. All animals were imaged 30 minutes post amputation. A representative close-up image is displayed in the panels (general ROS: green, hydrogen peroxide: yellow). Both stains (general ROS: carboxy-H_2DFCDA and hydrogen peroxide: PO1) are combined with a DPI-mediated ROS inhibition in order to check the specificity of the signal. Scale bar represents 100 µm.

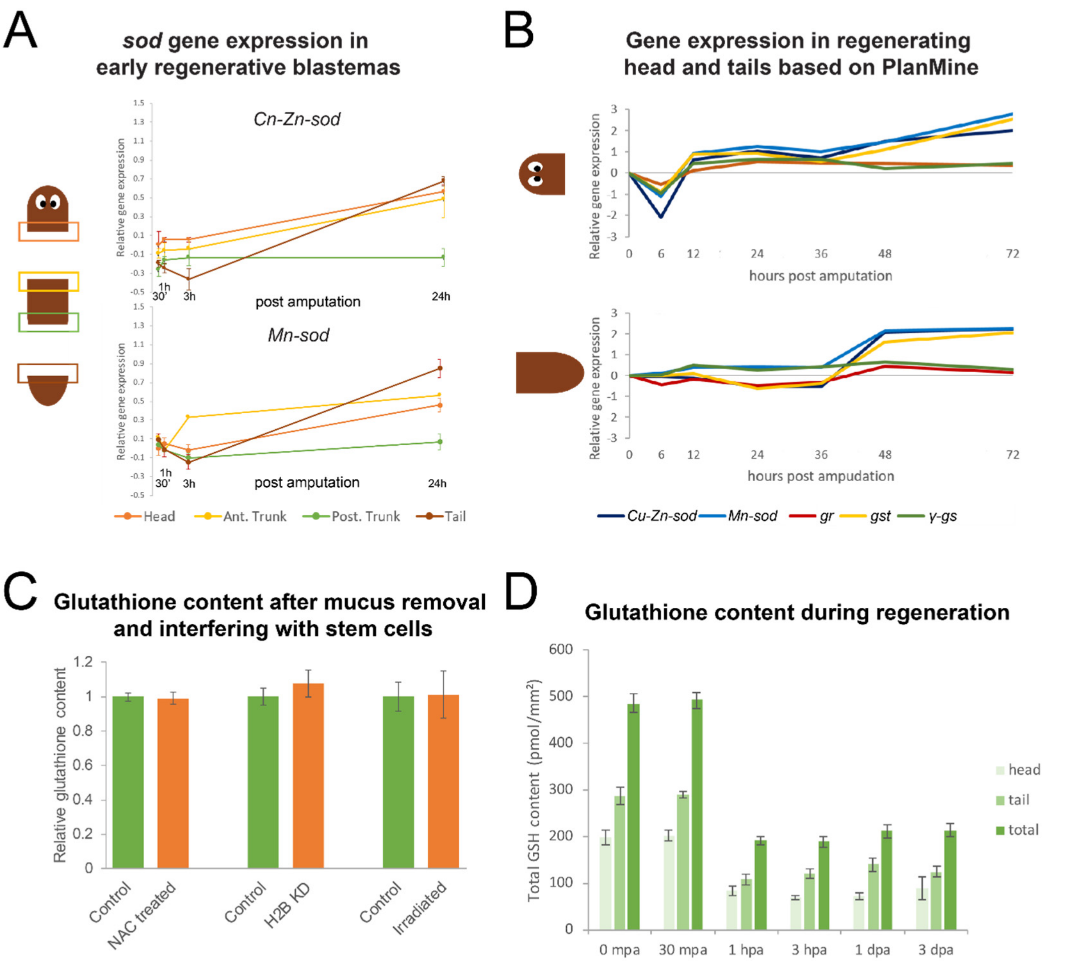

	30 mpa	1 hpa	3 hpa	1 dpa	3 dpa
0 mpa	ns	***	***	***	***
30 mpa		***	***	***	***
1 hpa			ns	ns	ns
3 hpa				ns	ns
1 dpa					ns

Figure S5 – Localisation of major antioxidant systems in the planarian body (continued). **A** Expression of *Cu-Zn-sod* and *Mn-sod* in head and tail blastemas during the first 24h of the regenerative process (n_{head} = 6, n_{tail} = 6, the error bars represent standard error). **B** Expression patterns of *Cu-Zn-sod*, *Mn-sod*, *gr*, *gst* and *γ-gs* in head and tail fragments during the first 72h of regeneration, based on transcriptome data deposited in PlanMine v3.0. **C** Total glutathione content in intact animals after removal of the mucus and interfering with the stem cells (n = 3, all ns, error bars represent standard error). **D** Total glutathione content in intact animals after removal of the mucus and interfering with the stem cells (n_{head} = 4, n_{tail} = 4, n_{total} = n_{head} + n_{tail}, error bars represent standard error). (*sod*: superoxide dismutase, *cat*: catalase, *gr*: glutathione reductase, *gst*: glutathione-S-transferase, *γ-gs*: γ-glutamylcysteine synthetase, *trx*: thioredoxin, mpa: minutes post amputation, hpa; hours post amputation, dpa: days post amputation, NAC: N-acetyl-L-cystein, KD: knockdown, n: number of observations, ns: not significant, *** $p < 0.001$)

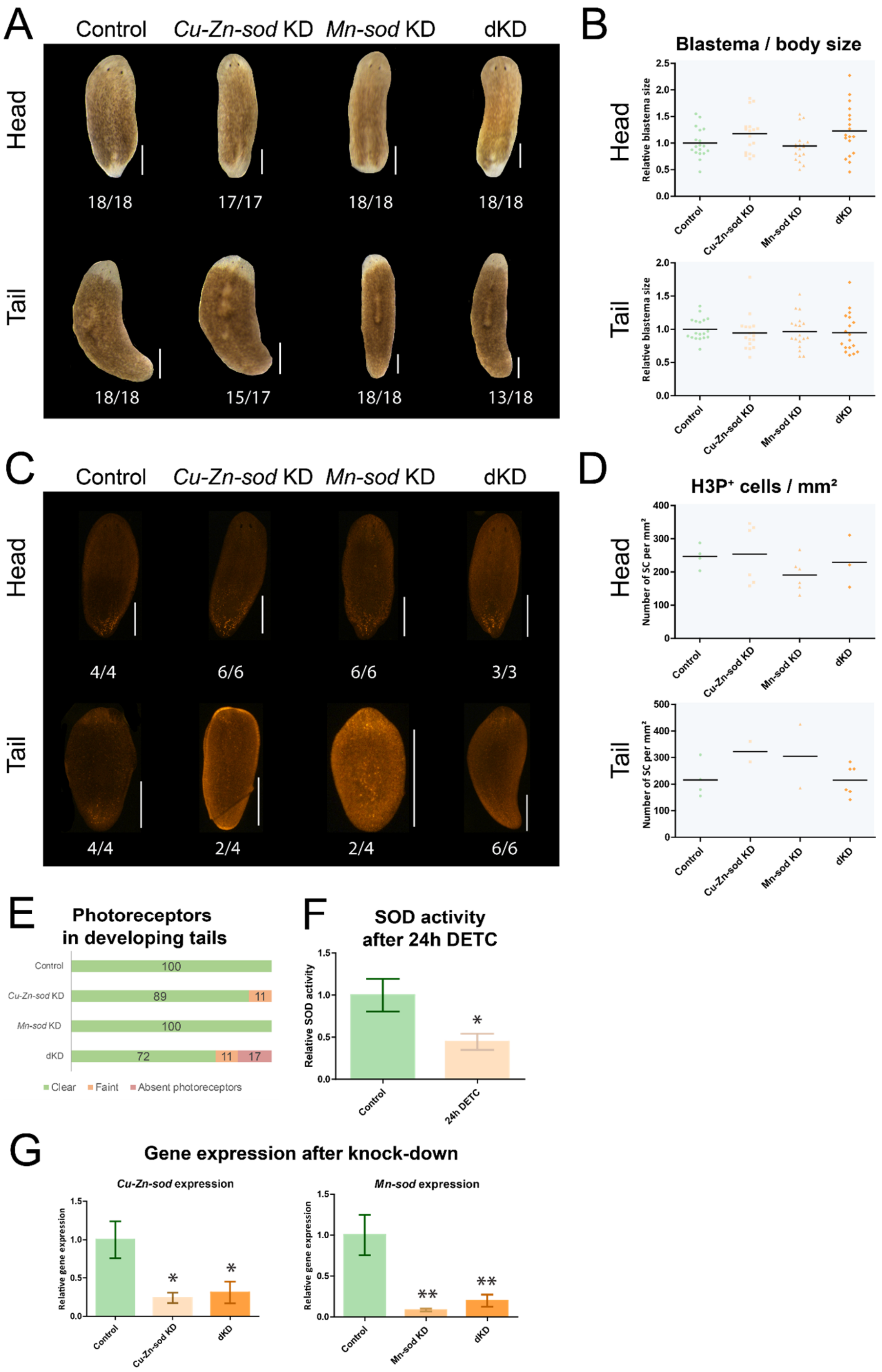

Figure S6 – Phenotypes after interfering with the expression of *Cu-Zn-sod* and *Mn-sod*. **A** Phenotypes after knockdown (KD) of *Cu-Zn-sod* and *Mn-sod*, 7 days after amputation. Both genes were targeted simultaneously, resulting in a double knockdown (dKD). The numbers below the worms represent how many times the depicted phenotype is observed versus the total number of observations (combined result of 2 experimental replicates). Scale bar represents 500 µm. **B** Blastema sizes normalized to the body size in head and tail fragments, relative to the control, 7 dpa (all ns, black line represents average per condition). **C** Histon H3P stain as measure of the number of proliferating stem cell in 7 dpa head and tail fragments. The numbers below the fragments represent how many times the depicted pattern is observed versus the total number of observations. Scale bar represents 500 µm. **D** Number of histon H3P⁺ cells, normalized over the surface area of the head or tail fragments, 7 dpa. (all compared to control: ns, black line represents average per condition). **E** Percentages of clear, faint or absent photoreceptors in 7 dpa tails. **F** Average SOD activity after 24h exposure to SOD inhibitor DETC ($n \geq 5$, * $p < 0.05$, error bars represent standard error). **G** Average expression levels of *Cu-Zn-sod* and *Mn-sod* measured by qPCR after KD. Head and tail fragments were pooled in the analysis. ($n = 6$, all compared to control: * $p < 0.05$, ** $p < 0.01$, error bars represent standard error). (dpa: days post amputation, ns: not significant)

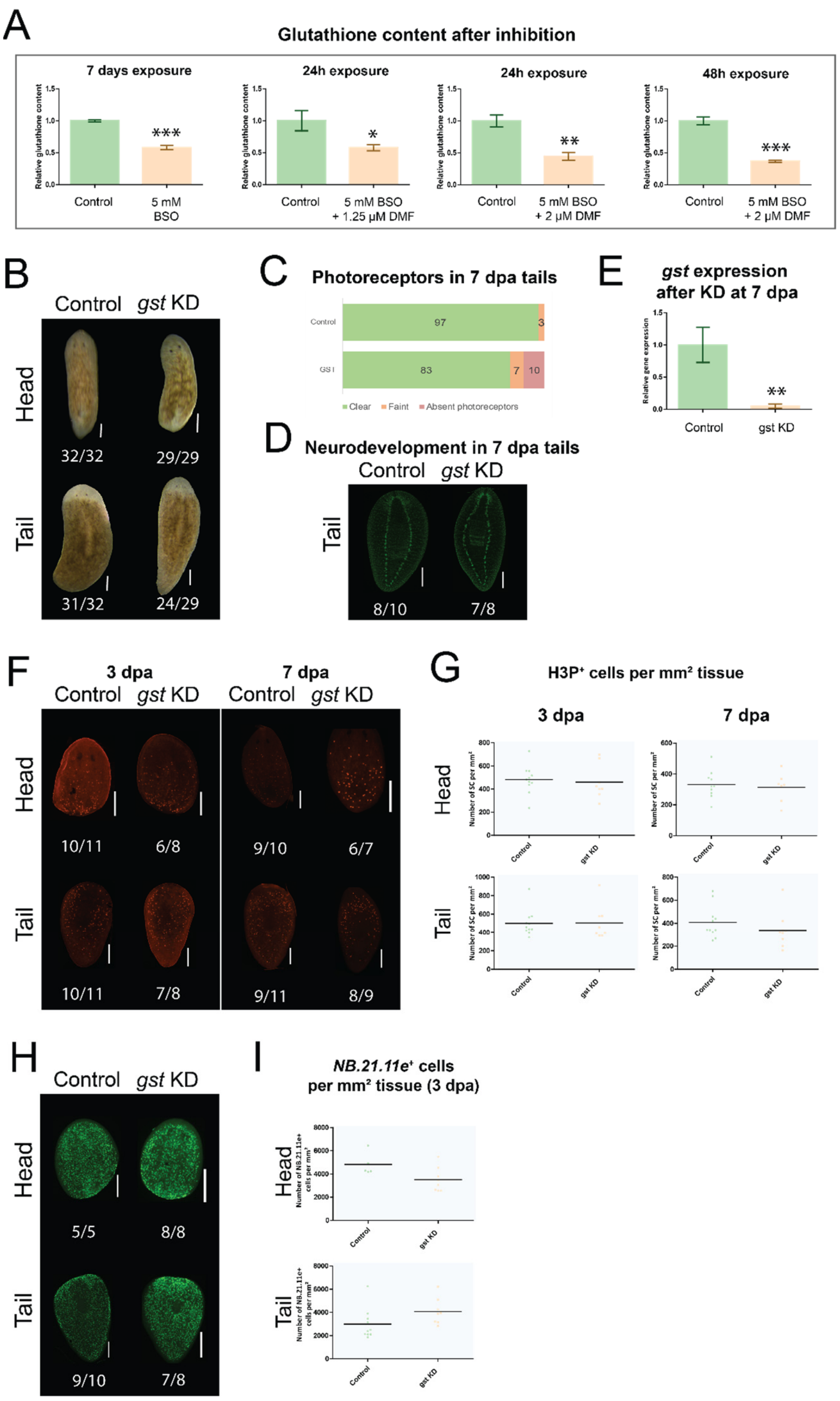

Figure S7 – Glutathione content after exposure to inhibitors and phenotypes and stem cell responses after interfering with the expression of *gst*. **A** Average glutathione content in intact animals after exposure to different concentrations of inhibitors during different exposure times. (n = 4, * $p < 0.05$, ** $p < 0.01$, *** $p < 0.001$, error bars represent standard error). **B** Phenotypes after knockdown (KD) of *gst*, 7 days after amputation. The numbers below the worms represent how many times the depicted phenotype is observed versus the total number of observations. Scale bar 500 µm. **C** Percentages of clear, faint or absent photoreceptors in 7 dpa tails. **D** Anti-synapsin stain after *gst* KD, 7 dpa. Scale bar represents 200 µm. **E** Average expression levels of *gst* measured by qPCR after KD, 7 dpa. Head and tail fragments were pooled in the analysis. (n = 6, compared to control: ** $p < 0.01$, error bars represent standard error). **F** Histon H3P stain as measure of the number of proliferating stem cell in 3 and 7 dpa head and tail fragments with a reduced *gst* expression. The numbers below the fragments represent how many times the depicted pattern is observed versus the total number of observations. Scale bar represents 200 µm. **G** Number of histon H3P^{+} cells, normalized over the surface area of the head or tail fragments, 3 and 7 dpa. (compared to control: ns, black line represents average per condition). **H** *NB.21.11e* stain as measure for the number of early progeny cells per mm² tissue in head and tail fragments, 3 dpa. Scale bar represents 200 µm. **I** Number of *NB.21.11e*$^{+}$ cells per mm² tissue in head and tail fragments, 3 dpa (compared to control: ns, black line represents average per condition). (BSO: buthionine sulphoximine, DMF: dimethyl fumarate, *gst*: glutathione-S-transferase, dpa: days post amputation)

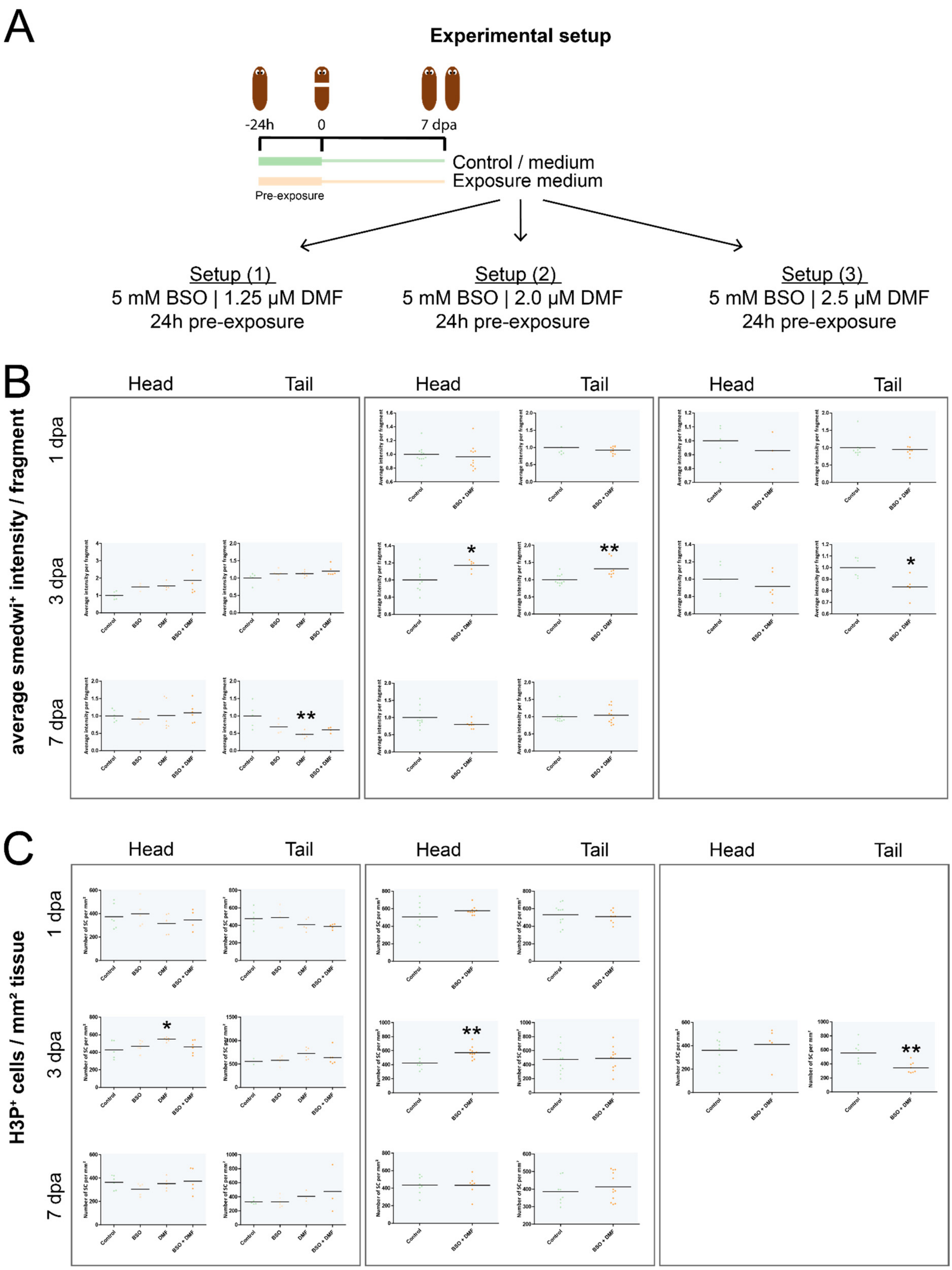

Figure S8 – Stem cell responses in regenerating worms after exposure to BSO and DMF in different concentrations. A Experimental setup: worms were pre-exposed for 24 hours to 5 mM BSO, 1.25–2.5 µM DMF or a combination of both. Next, they were amputated in head or tail fragments and were allowed to regenerate in the same exposure solutions. The number of (proliferating) stem cells were compared between the different groups in a total of 3 different setups, in both regenerating head and tail fragments, 1, 3 or 7 dpa. **B** Average intensity of *smedwi*$^+$ signal, relative compared to the control. ($n \geq 3$, all compared to control: * $p < 0.05$, ** $p < 0.01$, black line represents average per condition). **C** Number of histon H3P$^+$ cells, normalized over the surface area of the head or tail fragments. ($n \geq 3$, all compared to control: * $p < 0.05$, ** $p < 0.01$, black line represents average per condition). (BSO: buthionine sulphoximine, DMF: dimethyl fumarate, dpa: days post amputation)
